# Hospital readmission following acute illness among children 2–23 months old in sub-Saharan Africa and South Asia: a secondary analysis of CHAIN cohort

**DOI:** 10.1016/j.eclinm.2024.102676

**Published:** 2024-06-07

**Authors:** The Childhood Acute Illness and Nutrition (CHAIN) Network, Abdoulaye Hama Diallo, Abu Sadat Mohammad Sayeem Bin Shahid, Al Fazal Khan, Ali Faisal Saleem, Benson O. Singa, Blaise Siézan Gnoumou, Caroline Tigoi, Catherine Achieng, Celine Bourdon, Chris Oduol, Christina L. Lancioni, Christine Manyasi, Christine J. McGrath, Christopher Maronga, Christopher Lwanga, Daniella Brals, Dilruba Ahmed, Dinesh Mondal, Donna M. Denno, Dorothy I. Mangale, Emmanuel Chimezi, Emmie Mbale, Ezekiel Mupere, Gazi Md. Salauddin Mamun, Issaka Ouédraogo, James A. Berkley, Jenala Njirammadzi, John Mukisa, Johnstone Thitiri, Judd L. Walson, Julie Jemutai, Kirkby D. Tickell, Lubaba Shahrin, MacPherson Mallewa, Md. Iqbal Hossain, Mohammod Jobayer Chisti, Molly Timbwa, Moses Mburu, Moses M. Ngari, Narshion Ngao, Peace Aber, Philliness Prisca Harawa, Priya Sukhtankar, Robert H.J. Bandsma, Roseline Maïmouna Bamouni, Sassy Molyneux, Shalton Mwaringa, Shamsun Nahar Shaima, Syed Asad Ali, Syeda Momena Afsana, Syera Banu, Tahmeed Ahmed, Wieger P. Voskuijl, Zaubina Kazi

**Keywords:** Post-discharge, Children, Acute illness, Vulnerability, Low- and middle income

## Abstract

**Background:**

Children in low and middle-income countries remain vulnerable following hospital-discharge. We estimated the incidence and correlates of hospital readmission among young children admitted to nine hospitals in sub-Saharan Africa and South Asia.

**Methods:**

This was a secondary analysis of the CHAIN Network prospective cohort enrolled between 20th November 2016 and 31st January 2019. Children aged 2–23 months were eligible for enrolment, if admitted for an acute illness to one of the study hospitals. Exclusions were requiring immediate resuscitation, inability to tolerate oral feeds in their normal state of health, had suspected terminal illness, suspected chromosomal abnormality, trauma, admission for surgery, or their parent/caregiver was unwilling to participate and attend follow-up visits. Data from children discharged alive from the index admission were analysed for hospital readmission within 180-days from discharge. We examined ratios of readmission to post-discharge mortality rates. Using models with death as the competing event, we evaluated demographic, nutritional, clinical, and socioeconomic associations with readmission.

**Findings:**

Of 2874 children (1239 (43%) girls, median (IQR) age 10.8 (6.8–15.6) months), 655 readmission episodes occurred among 506 (18%) children (198 (39%) girls): 391 (14%) with one, and 115 (4%) with multiple readmissions, with a rate of: 41.0 (95% CI 38.0–44.3) readmissions/1000 child-months. Median time to readmission was 42 (IQR 15–93) days. 460/655 (70%) and 195/655 (30%) readmissions occurred at index study hospital and non-study hospitals respectively. One-third (N = 213/655, 33%) of readmissions occurred within 30 days of index discharge. Sites with fewest readmissions had the highest post-discharge mortality. Most readmissions to study hospitals (371/450, 81%) were for the same illness as the index admission. Age, prior hospitalisation, chronic conditions, illness severity, and maternal mental health score, but not sex, nutritional status, or physical access to healthcare, were associated with readmission.

**Interpretation:**

Readmissions may be appropriate and necessary to reduce post-discharge mortality in high mortality settings. Social and financial support, training on recognition of serious illness for caregivers, and improving discharge procedures, continuity of care and facilitation of readmission need to be tested in intervention studies. We propose the ratio of readmission to post-discharge mortality rates as a marker of overall post-discharge access and care.

**Funding:**

The 10.13039/100000865Bill & Melinda Gates Foundation (OPP1131320).

**Research in context**

**Evidence before this study**

We searched PubMed on 5th January 2024 using the following terms (“infant∗” [All Fields] OR “child∗” [All Fields] OR “paediatric∗” [All Fields] OR “pediatric∗” [All Fields]) AND (“readmission” [All Fields] OR “readmissions” [All Fields] OR “readmission∗” [All Fields] OR “readmit∗” [All Fields] OR “re admission∗” [All Fields] OR “rehospitali∗” [All Fields]) AND (“Africa∗” [All Fields] OR “Asia∗” [All Fields]) without language or publication date restrictions. Among general paediatric admissions, in a trial of azithromycin targeting post-discharge death or readmission in Kenya, 8.2% of children were readmitted during six months; and in Tanzania and Liberia reported 4.8% were readmitted during 60 days. Other relevant reports were for specific diseases (asthma, diabetes, HIV, sepsis, severe malnutrition, severe malaria, severe anaemia, acute asthma, and preterm neonates).

**Added value of this study**

In Africa and South Asia, 18% of acutely ill children admitted were readmitted during 6 months [41.0 (95% CI 38.0–44.3)/1000 child-months], with a different pattern over time to mortality. Age, prior hospitalisation, chronic conditions, illness severity, and maternal mental health were associated with readmission; however, sex, nutritional status, duration of hospitalisation, leaving hospital against medical advice and access to healthcare were not. Sites with fewest readmissions had the highest post-discharge mortality.

**Implications of all the available evidence**

Readmissions among children in low- and middle-income countries are common, predictable and have a different epidemiology to that of post-discharge mortality. We should improve discharge processes, and address families’ barriers to providing appropriate care and re-presenting to hospital when necessary.

## Introduction

Although there have been substantial reductions in childhood mortality over the past several decades, the number of child deaths remains high in sub-Saharan Africa and South Asia.^1^ Most of these deaths are caused by preventable and/or treatable infectious illnesses.^1^ Emerging evidence suggests that children in low-resource settings are particularly vulnerable following hospital-discharge.^2^, ^3^, ^4^, ^5^ A persistent risk of hospital readmission is recognised among adult and intensive care medical admissions, however, there remain significant gaps in our understanding of paediatric readmission in low- and middle-income countries (LMICs).^6^, ^7^, ^8^ Importantly, in the Childhood Acute Illness and Nutrition (CHAIN) Network cohort study, more than half of post-discharge deaths occurred at home rather than during a readmission.^3^

Prior paediatric studies reporting readmission have mostly focussed on specific diseases, a limited geographic scope, and/or have evaluated limited data on co-morbidities and socioeconomic exposures.^8^, ^9^, ^10^, ^11^ Among general paediatric admissions in Western Kenya, in a trial of azithromycin targeting to reduce post-discharge death or readmission, 8.2% of children were readmitted during six months.^12^ Azithromycin did not prevent death or readmission. A systematic review of six months readmission prevalence among children treated for severe anaemia in Africa, reported a pooled readmission frequency of 17%.^13^ Malaria chemoprevention, but not co-trimoxazole or micronutrient supplementation, has been shown to reduce incidence of mortality or readmission among children treated for severe anaemia in malaria endemic areas.^14^, ^15^, ^16^ Among HIV-negative children discharged from hospital following treatment for severe malnutrition in coastal Kenya and Nairobi, 20% were readmitted during twelve months, without significant protective efficacy of daily co-trimoxazole prophylaxis.^17^ A recent meta-analysis of these and other reports on post-discharge mortality, totalling 105,560 children from 46 cohorts, including the CHAIN cohort, identified disease subgroups of severe malnutrition and severe anaemia having the highest post-discharge mortality rates and unplanned discharges, severe malnutrition, and HIV seropositivity as risk factors.^5^ There has not been a systematic review of paediatric readmissions in LMICs.

In high-income settings, hospital readmission rates are used to measure quality of inpatient care, with national benchmarks for adult readmission rates.^8^^,^^18^ In such settings, 30-day reported readmission frequencies ranged widely from 3.4% to 29% across different disease conditions, with as many as a third of readmissions being deemed potentially preventable with well-planned inpatient-to-outpatient care transition and post-discharge support.^8^^,^^19^^,^^20^

Understanding the pattern and risks of readmission may help improve care at and after discharge in LMICs. We estimated the incidence of hospital readmission following index discharge, associated child and household characteristics, and examined ratios of readmission to post-discharge mortality rates among children aged 2–23 months discharged from hospital following acute illness across nine hospitals in six countries in sub-Saharan Africa and South Asia.

## Methods

### Study design

This was a secondary analysis of the prospective stratified CHAIN cohort.^3^ Children aged 2–23 months were recruited at admission to hospital for acute illness and followed up for six months after index discharge. For this analysis, the primary outcome was readmission to the study site or any other hospital during follow up.

### Setting

The CHAIN Network cohort study was conducted at nine hospitals in six countries across sub-Saharan Africa and South Asia reflecting a wide range of settings and endemic diseases. The hospitals were: Kilifi County, Mbagathi County and Migori sub-County Hospitals in Kenya; Mulago National Referral Hospital in Uganda, Queen Elizabeth Central Hospital, Blantyre in Malawi; Banfora Regional Referral Hospital in Burkina Faso; Civil Hospital, Karachi in Pakistan; and Dhaka and Matlab Hospitals in Bangladesh. Kilifi County, Migori sub-County, Banfora Regional and Matlab Hospitals were in rural settings, while the rest were in urban areas. Prior to the CHAIN cohort an assessment of the study hospitals including adherence to clinical care guidelines was conducted and support provided to meet guidelines as detailed elsewhere.^21^

### Participants

Children aged 2–23 months were eligible for enrolment, if admitted for an acute illness to one of the study hospitals. Exclusions were requiring immediate resuscitation, inability to tolerate oral feeds in their normal state of health, had suspected terminal illness, suspected chromosomal abnormality, trauma, admission for surgery, or their parent/caregiver was unwilling to participate and attend follow-up visits.^3^

The CHAIN cohort was stratified into three nutritional categories in ratio 2:1:2: a) Not wasted (NW): mid-upper arm circumference (MUAC) ≥12.5 cm at age ≥6 months or MUAC ≥12 cm at age <6 months old, b) Moderately wasted (MW): MUAC 11.5–<12.5 cm at age ≥6 months or MUAC 11.0 to <12.0 cm at age <6 months old, and c) Severely wasting/kwashiorkor (SWK): MUAC <11.5 cm at age ≥6 months or MUAC <11.0 cm at age <6 months old or kwashiorkor (nutritional oedema). Stratification was to ensure the study adequately represented the range of risks associated with nutritional status. During hospitalisation, children were treated following World Health Organisation (WHO) and national guidelines. At discharge, referral to available medical, nutrition and other services was undertaken. Decisions to admit and discharge children were made by the hospital clinicians independently of the study. Discharge data was collected after the decision to discharge was made. Scheduled study follow-up was at days 45, 90 and 180 after discharge.

### Data sources and measurement

At index admission and discharge, standardised data were collected covering child demographics; anthropometry; clinical symptoms and signs (Appendix p 3); complete blood count and laboratory tests for glucose, malaria, and HIV; caregiver characteristics, including maternal HIV status, mental health assessment using the PHQ9 questionnaire, employment and education; access to healthcare; and household characteristics including household composition, house construction, water & sanitation, household assets and food insecurity by trained study clinicians. Access to care was assessed by means of travel, travel time and cost of travel to the hospital, distance to the study hospital and distance to the nearest health facility as described in Appendix pp 4 and 5 and previously.^3^ At index discharge, a home visit was performed to verify household data collected in hospital and map the household location using GPS. Data on readmissions to study hospitals were documented on standardised proforma similar to the index admission and discharge assessment, including recording diagnoses and comorbidities. During scheduled study follow-up at days 45, 90 and 180, data on readmissions to non-study hospitals were collected, including date of readmission and name of the hospital, however, readmission diagnosis were not systematically or reliably available for non-study hospital readmissions. All data collection tools are available at https://chainnetwork.org/resources and detailed further in the primary publication on mortality.^3^

In this secondary analysis, the primary outcome was hospital readmission after index discharge. Some children had multiple readmissions; therefore, readmission was analysed as a multiple event. Demographic, anthropometric, clinical, maternal, household and socioeconomic variables were defined as detailed in the Appendix pp 3–6 and previously.^3^ As previously, individual variables were grouped a priori into domains and categorised into tertiles for regression modelling.^3^ Briefly, there were seven domains: underlying medical conditions, child-level nutritional risk exposures, signs of illness severity at admission, signs of illness severity at discharge, access to health care, household-level exposures, and caregiver characteristics. Individual variables in each domain are provided in the Appendix pp 5 and 6 and previously.^3^

### Ethics

The CHAIN cohort study and subsequent analyses were approved by the University of Oxford Tropical Research Ethics Committee in UK (OxTREC 34–16), and research ethical committees in each participating country.^3^^,^^22^ Caregivers of all participating children provided written informed consent.

### Statistics

Number of readmissions episodes was reported because some children had multiple readmissions. Baseline characteristics were reported stratified by any readmission and no readmissions. Time to events was calculated per 1000 child-months from date of index discharge to date(s) of readmission(s), death, lost to follow-up (LTFU), or 180 days later. Readmissions within month one after index discharge were considered as early readmissions and the rest as late readmissions. Early and late hospital readmission rates were compared using rate ratios adjusted for age, sex and the hospital site. To examine correlates of post-discharge mortality, the ratio of hospital readmission to post-discharge mortality rates were calculated for each site, per month following discharge, per age group, and per nutritional strata, and tested for heterogeneity across these groups using random-effects meta-analysis. A ratio of one would mean one readmission for each post-discharge death while a ratio greater than one means more readmissions per post-discharge death. We formally tested the hypothesis that groups with high readmission rates would have low post-discharge mortality rates by performing a linear regression of non-fatal hospital readmissions (to avoid overlap of readmissions that resulted to death) versus post-discharge mortality rates across sites, nutritional strata, follow-up month and age group with appropriate random effects.

To examine individual factors and constructed domains' associations with hospital readmission, multilevel survival regression models accounting for heterogeneity by site and multiple readmissions per child, were undertaken. We used the Fine and Gray competing risk regression model accounting for the recruiting hospital heterogeneity and allowing recurrent readmissions episodes implemented in the tidycmprsk R package (https://mskcc-epi-bio.github.io/tidycmprsk/). To account for the cohort stratification, sampling weights, calculated as explained elsewhere, were applied to the regression models.^3^ Model performance was assessed using bootstrapped area under the receiver operating curves (AUROC) with a probit model, resampled 1000 times with replacement. The measure of effect from the multilevel competing risk model were sub-distribution hazard ratio (SHR). As sensitivity analyses, to understand individual variables within domains, we performed an ‘exploded’ multivariable competing risk regression including the individual variables rather than the domain variables. Statistical analyses were conducted using Stata (Version 17.0, StataCorp, College Station, TX, USA) and R (version 4.2.0).

The original CHAIN cohort study was designed with 80% power to detect differences in proportion of children who would die post-discharge between non-wasted and moderately wasted children, with two-tailed α = 0.05 and 10% loss to follow-up. This secondary analysis used data from all the 2874 children discharged alive and followed up. Since readmissions were more common (18%) than post-discharge mortality (5.8%), this secondary analysis was considered adequately powered.

### Role of the funding source

The funder had no role in the study design, data collection, analyses, and interpretation, writing of the report or decision to submit the manuscript for publication.

## Results

Of the 2874 children enrolled in CHAIN cohort and discharged alive from their index admission (1239 (43%) girls, median (IQR) age 10.8 (6.8–15.6) months), there were 655 readmissions among 506 (18%, 95% CI 16–19%) children (198 (39%) girls): 391 (77%) had one readmission, 91 (18%), 17 (3.4%), 4 (0.8%) and 3 (0.6%) had two, three, four and five readmissions respectively. Of the 655 episodes, 460 (70%) occurred at the index study hospital and 195 (30%) at non-study hospitals. Among the 460 episodes at the index study hospital 6/460 (1.3%) occurred during a scheduled study follow-up visit. Table 1 and Appendix p 7 shows baseline characteristics of study participants stratified by readmission status.

**Table 1:** Child characteristics at index admission and discharge.

	All participants (N = 2874)	No readmission (N = 2368)	At least one readmission (N = 506)	P-value
**Demographics**				
Cohort strata				
Not wasted (NW)	1072 (37)	899 (38)	173 (34)	0.23
Moderately wasted (MW)	724 (25)	595 (25)	129 (26)	
Severely wasted/kwashiorkor (SKW)	1078 (38)	874 (37)	204 (40)	
Age — months median (IQR)	10.8 (6.8–15.6)	11.0 (7.0–15.8)	10.3 (6.2–14.8)	0.01
Sex — no. (%)				
Male	1635 (57)	1327 (56)	308 (61)	0.05
Female	1239 (43)	1041 (44)	198 (39)	
Recruiting site				
Kilifi County Hospital, Kenya	227 (7.9)	187 (7.9)	40 (7.9)	<0.0001
Mbagathi Hospital, Kenya	254 (8.8)	199 (8.4)	55 (11)	
Migori County Hospital, Kenya	233 (8.1)	209 (8.8)	24 (4.7)	
Mulago National Rereferral Hospital, Uganda	446 (16)	369 (16)	77 (15)	
Queen Elizabeth Central Hospital, Malawi	285 (9.9)	220 (9.3)	65 (13)	
Dhaka Hospital, Bangladesh	385 (13)	291 (12)	94 (19)	
Matlab Hospital, Bangladesh	311 (11	270 (11)	41 (8.1)	
Karachi Civil Hospital, Pakistan	337 (12)	272 (11)	65 (13)	
Banfora Regional Referral Hospital, Burkina Faso	396 (14)	351 (15)	45 (8.9)	
Length of admission — days median (IQR)	4 (2–7)	4 (2–7)	5 (3–8)	0.02
Discharge on a weekend — no. (%)	572 (20)	467 (20)	105 (21)	0.60
Leaving against medical advice	205 (7.1)	177 (7.5)	28 (5.5)	0.12
Change in anthropometry at hospital discharge^a^				
No change	2447 (85)	2014 (85)	433 (86)	0.02
Improved	354 (12)	302 (13)	52 (10)	
Worsened	73 (2.5)	52 (2.2)	21 (4.2)	
**Clinical presentation at admission**				
SIRS — no. (%)^b^	948 (33)	747 (32)	201 (40)	<0.0001
Severe pneumonia — no. (%)^c^	589 (20)	462 (20)	127 (25)	0.005
Diarrhoea — no. (%)	1580 (55)	1317 (56)	263 (52)	0.14
Malaria (RDT positive) — no. (%)	408 (14)	350 (15)	58 (11)	0.008
Anaemia — no. (%)^d^				
None	558 (19)	458 (19)	100 (20)	0.90
Mild	652 (23)	532 (22)	120 (24)	
Moderate	1261 (44)	1046 (44)	215 (42)	
Severe	403 (14)	332 (14)	71 (14)	
Blood glucose — no. (%)^e^				
Normal blood glucose	2674 (93)	2202 (93)	472 (93)	0.82
Abnormal blood glucose	200 (7.0)	166 (7.0)	34 (6.7)	
**Acute illness at index discharge**				
SIRS — no. (%)^b^	415 (14)	338 (14)	77 (15)	0.58
Severe pneumonia — no. (%)^c^	167 (5.8)	140 (5.9)	27 (5.3)	0.62
Anaemia — no. (%)^d^				
None	357 (12)	279 (12)	78 (15)	0.03
Mild	711 (25)	607 (26)	104 (21)	
Moderate	1586 (55)	1302 (55)	284 (56)	
Severe	220 (7.7)	180 (7.6)	40 (7.9)	
**HIV status**				
**HIV status — no. (%)**				
Negative	2570 (89)	2138 (90)	432 (85)	0.001
HIV infected	77 (2.7)	45 (1.9)	24 (4.7)	
HIV exposed	158 (5.5)	124 (5.2)	34 (6.7)	
Refused/untested	69 (2.4)	61 (2.6)	16 (3.2)	
**Underlying conditions**				
Stunting — no. (%)^f^				
None	1469 (51)	1239 (52)	230 (45)	<0.0001
Moderate	665 (23)	554 (23)	111 (22)	
Severe	740 (26)	575 (24)	165 (33)	
Small birth size — no. (%)^g^	482 (17)	387 (16)	95 (19)	0.18
Chronic conditions — no. (%)^h^	192 (6.7)	130 (5.5)	62 (12)	<0.0001
Prior hospitalization — no. (%)				
No prior admission	2140 (74)	1821 (77)	319 (63)	<0.0001
Prior admission	734 (26)	547 (23)	187 (37)	
**Caregiver characteristics**				
Biological mother is primary caregiver — no. (%)	2743 (95)	2263 (96)	480 (95)	0.49
Mother currently sick — no. (%)	426 (15)	348 (15)	78 (15)	0.68
Caregiver education — no. (%)				
None	749 (26)	624 (26)	125 (25)	0.16
Primary	1228 (43)	1023 (43)	205 (41)	
Secondary/Tertiary	897 (31)	721 (30)	176 (35)	
Maternal mental health (PHQ9) score — no. (%)				
None to mild	2338 (81)	1957 (83)	381 (75)	<0.0001
Moderate to severe	536 (19)	411 (17)	125 (25)	
Mother working — no. (%)				
Self-employed	570 (20)	469 (20)	101 (20)	0.29
Employed	282 (9.8)	223 (9.4)	59 (12)	
No reported income	2022 (70)	1676 (71)	346 (68)	
**Household-level exposures**				
Assets index — no. (%)				
Quintile 1 (Least assets)	541 (19)	462 (20)	79 (16)	0.02
Quintile 2	553 (19)	471 (20)	82 (16)	
Quintile 3	570 (20)	469 (20)	101 (20)	
Quintile 4	598 (21)	476 (20)	122 (24)	
Quintile 5 (Most assets)	612 (21)	490 (21)	122 (24)	
Household food insecurity — no. (%)				
Low	1745 (61)	1465 (62)	280 (55)	0.004
Medium	776 (27)	632 (27)	144 (28)	
High	353 (12)	271 (11)	82 (16)	
**Access to health care**				
Distance to study hospital (km) median (IQR)	8.94 (4.40–19.7)	9.28 (4.51–21.0)	7.78 (4.06–14.5)	0.002
Distance to nearest health facility (km) median (IQR)	1.26 (0.52–3.09)	1.31 (0.55–3.24)	1.05 (0.44–2.56)	0.003

Data are N (%) or median (IQR), P-values are from chi-square or Wilcoxon Rank Sum tests.

Abbreviation: IQR, Interquartile range; SIRS, Systemic Inflammatory Response Syndrome; RDT, rapid diagnostic test.

a Change in anthropometry was estimated using Mid-Upper Arm circumference (MUAC), improved means discharge MUAC was greater than admission MUAC, while worsen means the MUAC declined at discharge.

b SIRS defined as presence of two of the following four criteria; heart rate low (<90) or high (>180)/min; temperature low (<36 °C) or high (≥38.5 °C); respiratory rate high (>34 breaths per minute) and WBC low (<5 × 10^9^/l) or high (>17.5 × 10^9^/l).

c Severe pneumonia: Cough or difficulty in breathing with; oxygen saturation <90% or central cyanosis, or grunting, very severe chest indrawing, or inability to breastfeed or drink, lethargy or reduced level of consciousness, convulsions.

d Anaemia by haemoglobin defined as None: >11, Mild: 10–11, Moderate: 7–10 and Severe: <7.0 g/dl.

e Abnormal blood glucose defined as blood glucose <3 or 10 mmol/l.

f Stunting: none, length-for-age z score ≥−2; moderate, length-for-age z score −2 to −3; severe, length-for-age z score <−3.

g Small birth size was defined as reported low birth weight (<2.5 kg) or born premature.

h Chronic conditions includes thalassemia, cerebral palsy, sickle cell disease, congenital cardiac disease and known TB.

**Fig. 1:**
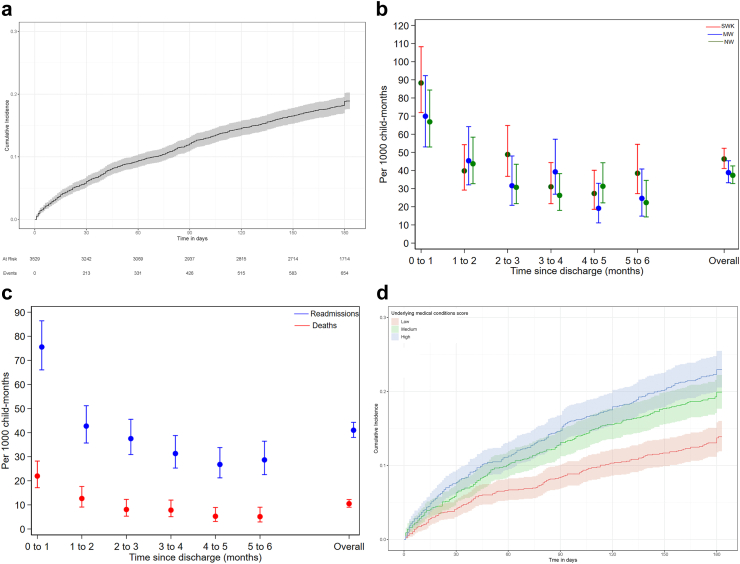
**a) Cumulative incidence of hospital readmission, b) Monthly readmission rates stratified by cohort strata, c) Monthly hospital readmission and post-discharge mortality rates and d) Cumulative hazard of readmission by underlying medical conditions domain score**. The error bars or shaded regions show 95% confidence Intervals.

The 655 hospital readmission episodes were observed over 15,971 child-months: 41.0 (95% CI 38.0–44.3) readmissions/1000 child-months (Fig. 1a). The median time to hospital readmission was 42 (IQR 15–93) days. One-third (N = 213/655, 33%) and two-thirds (N = 433/655, 66%) of readmissions occurred within one and three months after index discharge, respectively (Appendix pp 8, 18). Fig. 1b and Appendix p 9 show the monthly readmission rates by cohort strata. The monthly readmission rate was highest in the first month after the index discharge: 75.6 (95% CI 66.1–86.4)/1000 child-months and was 28.7 (95% CI 22.6–33.8)/1000 child-months during the last month of follow-up, with no linear trend (P = 0.16) (Fig. 1c & Appendix p 9). The risk of early readmission (first month) was significantly higher than late readmission: age, sex and site adjusted rate ratio 2.01 (95% CI 1.70–2.37) and was similar across cohort strata (Appendix pp 8). Fig. 1c shows the trend in monthly hospital readmissions and post-discharge mortality rates. Readmission rates were highest among children with HIV and chronic infections at index admission Appendix pp 9, 19 and 20.

**Fig. 2:**
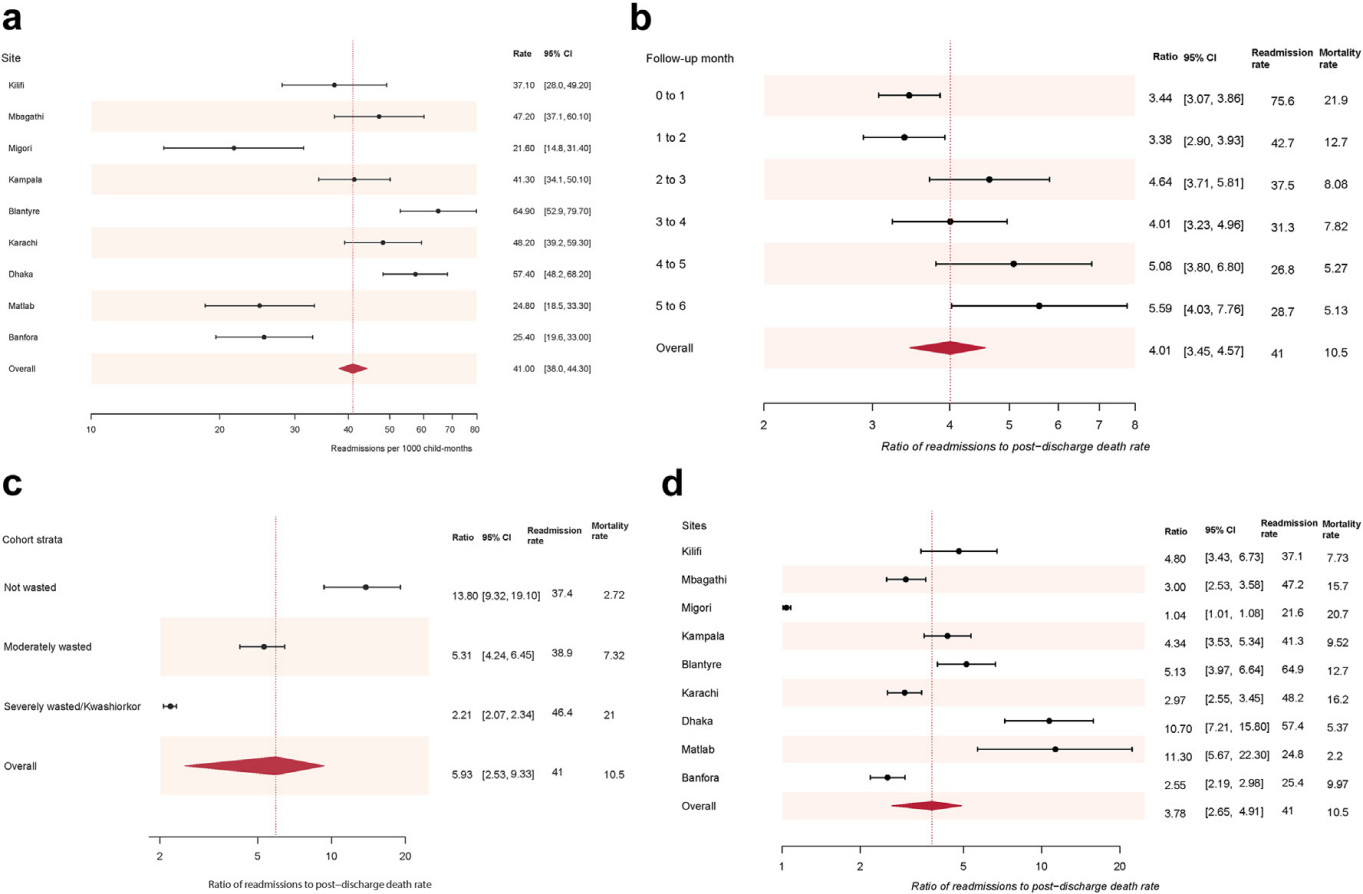
**a) Site readmission rates, per 1000 child-months, and ratios of hospital readmission to post-discharge mortality rates stratified by b) monthly follow-up, c) Cohort strata, and d) Site**. Data are readmission rate, or readmission rate to mortality rate ratio, with 95% CI, CI; Confidence Intervals, the dotted lines show the overall point readmission rate or readmission rate to mortality rate ratio and the diamonds show their 95% CI.

Readmission rates varied across sites, it was highest in Blantyre, Malawi (64.9 (95% CI 52.9–79.7)/1000 child-months) followed by Dhaka, Bangladesh (57.4 (95% CI 48.2–68.2)/1000 child-months) sites and lowest in Migori, Kenya (21.6 (95% CI 14.8–31.4)/1000 child-months), heterogeneity test P < 0.0001 (Appendix p 10 & Fig. 2a). The site-specific readmission and post-discharge mortality rates are shown in Appendix p 10.

### Diagnoses among readmissions to study hospitals

Among the 460 children readmitted to study hospitals, clinical characteristics at readmission are shown in Appendix pp 11 and 12. The leading readmission diagnoses were pneumonia (N = 255, 55%), gastroenteritis (N = 146, 32%), sepsis (N = 91, 20%), anaemia (N = 81, 18%) and malaria (N = 56, 12%) (Table 2). 371/460 (81%) readmissions to study hospitals had the same diagnosis recorded as during index admission: pneumonia 145/202 (72%), anaemia 47/88 (53%), malaria 31/62 (50%), sepsis 35/76 (46%), and gastroenteritis 77/184 (42%) (Appendix p 13).

**Table 2:** Most frequent diagnoses assigned by clinician during readmission to a study hospital.

Readmission diagnosis	Readmission to study hospitals <1 month after discharge (N = 153)		Readmissions to study hospitals ≥1 month after discharge (N = 307)		All readmissions to study hospitals (N = 460)	
	N	% (95% CI)	N	% (95% CI)	N	% (95% CI)
Pneumonia	83	54 (46–62)	172	56 (50–62)	255	55 (51–60)
Gastroenteritis	54	35 (28–43)	92	30 (25–35)	146	32 (28–36)
Sepsis	41	27 (20–35)	50	16 (12–21)	91	20 (16–24)
Anaemia	23	15 (9.8–22)	58	19 (15–24)	81	18 (14–21)
Malaria	8	5.2 (2.3–10)	48	16 (12–20)	56	12 (9.3–16)
Bronchiolitis	9	5.9 (2.7–11)	21	6.8 (4.3–10)	30	6.5 (4.4–9.2)
Measles	13	8.5 (4.6–14)	9	2.9 (1.3–5.5)	22	4.8 (3.0–7.2)
URTI	6	3.9 (1.5–8.3)	14	4.6 (2.5–7.5)	20	4.3 (2.7–6.6)
Pulmonary TB	6	3.9 (1.5–8.3)	13	4.2 (2.2–7.1)	19	4.1 (2.5–6.4)
Sickle cell disease	3	2.0 (0.4–5.6)	11	3.6 (1.8–6.3)	14	3.0 (1.7–5.1)
Cerebral palsy	4	2.6 (0.7–6.6)	7	2.3 (0.9–4.6)	11	2.4 (1.1–4.2)
Probable meningitis	5	3.3 (1.1–7.5)	5	1.6 (0.5–3.8)	10	2.2 (1.0–4.0)

Data are N (%, 95% confidence intervals), CI; Confidence intervals, a child can have more than one diagnosis, URTI, upper respiratory tract infection; TB, Tuberculosis.

### Deaths during readmission

Overall, 66/655 (10%, 95% CI 8.0–13%) readmissions resulted in inpatient death. Risk of death during readmission was similar between early readmissions (23/213 (11%, 95% CI 7.0–16%)) and late readmissions (43/442 (9.7%, 95% CI 7.1–13%)); P = 0.40 (Appendix p 18).

### Ratio of rates of readmission to post-discharge mortality

The pooled ratio of readmission to mortality rates across the groups examined ranged from 3.78 (95% CI 2.65–4.91) to 5.93 (95% CI 2.53–9.33) across the sites and nutrition strata respectively (Fig. 2b, c and d). Across the follow-up months, the readmission to mortality rate ratios varied from 3.44 (95% CI 3.07–3.86) between index discharge and month one to 5.59 (95% CI 4.03–7.76) between months five and six, (I-squared = 63.9%, P = 0.017) (Fig. 2b). The ratio of readmission to post-discharge mortality rates was highest among children not wasted at index admission (more readmissions per death) at 13.8, 95% CI 9.32–19.1, and lowest among severely malnourished children at index admission (fewer readmissions per death) at 2.21, 95% CI 2.07–2.34 (I-squared = 96.1%, P < 0.0001) (Appendix p 13 & Fig. 2c). The readmission to mortality rate ratio was lowest among infants <6 months old 3.13 (95% CI 2.77–3.54) and highest among children ≥12 months, (I-squared = 87.2%, P < 0.0001) (Appendix pp 10, 21).

Across sites, the readmission to mortality rate ratios varied from 1.04 (95% CI 1.01–1.08) in Migori to 11.3 (95% CI 5.67–22.3) in Matlab, (I-squared = 97.4%, P < 0.0001) (Fig. 2d & Appendix pp 10, 21). In a linear regression model, the non-fatal readmission rate was inversely associated with post-discharge mortality rate across sites (coefficient −0.33, 95% CI −0.41 to −0.25; P < 0.0001) whilst across follow up months, age groups and nutritional strata this was positively associated (Appendix p 21).

### Factors associated with hospital readmission

Factors associated with hospital readmission in univariate models are shown in Appendix pp 14 and 15. In the multivariable model increasing age in months (log transformed) was associated with lower risk of readmission (aSHR 0.84 (95% CI 0.74–0.96). High illness severity (aSHR 1.33 (95% CI 1.09–1.63)) at the time of index admission was associated with hospital readmission but not illness severity at index discharge (aSHR 0.71 (95% CI 0.36–1.42)). Untested HIV status was also associated with hospital readmission (aSHR 1.66 (95% CI 1.11–2.49)), while being HIV exposed or infected were not. Medium and high levels of underlying medical conditions were positively associated with readmission: (aSHR 1.42 (95% CI 1.15–1.75)) and (aSHR 1.59 (95% CI 1.26–1.99)), respectively (Fig. 1d). Most adverse caregiver characteristics (aSHR 1.30 (95% CI 1.07–1.58)) was associated with hospital readmission. However, access to health care, nutritional status at index admission, changes in anthropometry between index admission and discharge, household level exposures domain, length of index hospitalisation, sex, having left hospital against medical advice and index discharge on a weekend were not associated with readmission (Table 3). The multivariable model bootstrapped AUC was 0.81 (95% CI 0.80–0.82). Multivariable regression models with individual variables are shown in Appendix pp 15–17.

**Table 3:** Characteristics associated with hospital readmission.

	Readmission episodes (N = 655)	aSHR (95% CI)^a^	P-value
Age months (log)	–	0.85 (0.75–0.97)	0.02
Sex; female	259 (40)	0.91 (0.78–1.07)	0.34
Left hospital against medical advice	34 (5.2)	0.72 (0.49–1.07)	0.10
Admission duration days (log)	–	1.07 (0.93–1.22)	0.31
Index discharge on a weekend	137 (21)	0.99 (0.81–1.20)	0.94
**Cohort nutritional status strata**			
Not wasted	228 (35)	Reference	
Moderately wasted	159 (24)	0.98 (0.78–1.22)	0.90
Severely wasted or kwashiorkor	268 (41)	1.01 (0.79–1.30)	0.96
**Change in anthropometry from admission to discharge**			
No change	561 (86)	Reference	
Improved	67 (10)	0.85 (0.65–1.12)	0.31
Worsened	27 (4.1)	1.45 (0.96–2.18)	0.08
**Signs of illness severity at admission**			
Low	199 (30)	Reference	
Medium	204 (31)	1.16 (0.95–1.42)	0.24
High	252 (39)	1.33 (1.09–1.63)	0.005
**Signs of illness severity at discharge**			
Low	547 (84)	Reference	
Medium	99 (15)	0.98 (0.78–1.23)	0.95
High	9 (1.4)	0.71 (0.36–1.42)	0.30
**HIV status**			
Negative	559 (85)	Reference	
Untested	29 (4.4)	1.65 (1.11–2.46)	0.01
Exposed	50 (7.6)	1.32 (0.97–1.80)	0.07
Infected	17 (2.6)	0.89 (0.55–1.46)	0.70
**Underlying medical conditions**			
Low	157 (24)	Reference	
Medium	239 (36)	1.43 (1.16–1.76)	<0.0001
High	259 (40)	1.58 (1.26–1.98)	<0.0001
**Child-level nutritional risk exposures**			
Low	382 (59)	Reference	
Medium	68 (10)	1.02 (0.79–1.33)	0.90
High	205 (31)	1.01 (0.82–1.25)	0.97
**Caregiver characteristics**			
Least adverse	231 (35)	Reference	
Moderately adverse	220 (34)	1.16 (0.95–1.42)	0.14
Most adverse	204 (31)	1.29 (1.04–1.58)	0.02
**Household-level exposures**			
Least adverse	277 (42)	Reference	
Moderately adverse	200 (31)	0.90 (0.73–1.10)	0.31
Most adverse	178 (27)	0.87 (0.69–1.10)	0.20
**Access to health care**			
Least adverse	236 (36)	Reference	
Moderately adverse	232 (35)	1.07 (0.89–1.29)	0.54
Most adverse	187 (29)	1.07 (0.85–1.35)	0.50
Bootstrapped AUC (95% CI)		0.81 (0.80–0.82)	

Readmission episodes are reported as N (%) for categorical exposures.

All model results were weighted using sampling and lost to follow up weights.

SHR and P-values from multivariable competing risk survival model with site as random effect.

a aSHR-adjusted Sub-distribution Hazard ratios for all predictors in the multivariable model, AUC; area under receiver operating characteristic curve, CI; Confidence Interval.

## Discussion

The present study shows that almost one-fifth (18%) of children, discharged after a hospital admission for an acute illness in sub-Saharan Africa and South Asia had at least one readmission more often for recurrent infection. There was significant heterogeneity in readmission across sites, likely reflecting differences in patient profile, healthcare quality and accessibility.^3^^,^^21^ There was no significant difference in readmission rates between children by nutritional strata, unlike the strong effect observed for mortality.^2^^,^^3^^,^^23^ Malnourished children had a low ratio of readmission to post-discharge deaths, suggesting financial and cultural barriers to readmission, particularly given that physical healthcare access score was not associated with readmission. This is supported by findings of a parallel qualitative study revealing healthcare costs, stigma by healthcare workers, little continuity of care, and maternal limited agency in decision making regarding medical treatment in some settings.^24^^,^^25^

Heterogeneity between sites is not surprising as sites differ in patient profiles, geography, healthcare access, socioeconomics and cultural factors.^22^ The two Bangladeshi sites were notable for the highest ratio of readmission to post-discharge mortality rates and among the lowest overall mortality. Admissions were predominantly for gastroenteritis and both hospitals provide clinical care for free, unlike other sites that levy user fees and/or costs of treatments and investigations. The International Centre for Diarrhoeal Disease Research, Bangladesh (ICDDR,B) has invested in building relationships with communities and parents facilitating returning to the hospital in case of health concerns. This ‘connectedness’ to the health system as well as the patient disease profile meant fewer post-discharge deaths.^24^

In high-income settings, reduction of both readmission and post-discharge mortality are commonly targeted together.^20^^,^^26^ In our setting, some readmissions are likely to be consequences of inadequate diagnosis and treatment during the index admission. However, our observation of sites with highest post-discharge mortality having fewest readmissions and the inverse relationship indicates likely inadequate readmission rates and thus barriers to readmission need to be addressed. In our concurrent qualitative work and similar study in Uganda, costs of the index admission profoundly impacted households’ financial and social capital, making attendance to a follow-up clinic, purchase of take home medication or another admission more difficult for vulnerable families.^24^^,^^27^ In high mortality settings, underlying factors such as severe malnutrition, HIV or anaemia cannot be fully remedied during a short hospital admission making a new episode of illness far more likely than in high-income settings,^5^ thus increasing appropriate readmission may be desirable to reduce mortality. This may involve both health system changes and support for families.

Older children had lower risk of readmission, as was seen for mortality, however, the young infants <6 months old who had highest risk of mortality had the lowest readmission to mortality rate ratio.^3^ This is likely a reflection of the complex and greater vulnerability among younger children and difficulty interpreting clinical symptoms and signs, thus emphasising a specific need to improve discharge processes in infants, for example to include information to mothers on danger signs.^8^ Maternal PHQ9 score, a screening tool for depression, which may indicate a mother's ability to meet her child's needs,^28^ was also associated with readmission.

Currently, there is minimal focus on discharge practices in WHO (including hospital and Integrated Management of Childhood Illness), national, or local guidelines,^29^ potentially contributing to weak discharge processes.^4^ Discharge decisions are commonly made by the least experienced staff members, based on their assessment, caregiver's request or need to create more bed space in the wards.^27^^,^^30^ Thus, poorly-planned discharge processes may send home children who are incompletely treated with high risk of post-discharge mortality.^5^ Some of these continuing illness episodes could be picked up by early post-discharge follow-up. However, the key point regarding longer-term mortality risk is that during and after an admission for acute illness, no changes have been made to longer term underlying child-, maternal- and household-level vulnerabilities. Currently, post-discharge review after acute illness depends on clinician judgement and ability of the caregiver to access health care.^24^^,^^27^ Our findings, and recent data from Uganda, show both mortality and readmission following index discharge can be predicted as accurately as inpatient deaths.^3^^,^^4^^,^^31^ However, improvements will need implementation of specific care pathways rather than a just a simple predictive tool. Beyond identifying children who are younger and more severely ill at index admission, we show that chronic medical conditions, repeated admissions, family finances, and maternal physical and mental health are important.

Future research should focus on identifying appropriate management changes in response to risk assessment, raising awareness among health providers of post-discharge mortality and readmission risks; improving communication, discharge processes, and care continuity between hospitals and community providers^2^^,^^23^^,^^32^; improving parental knowledge of recognising a sick child at home; facilitating readmissions (transport and prioritising assessment of recently discharged children); reducing or mitigating the costs to families of admission and follow up; and addressing maternal physical and mental health. The inverse relationship readmission and post-discharge mortality, and the varied drivers of readmission mean that a combined endpoint of death or readmission unsatisfactory for clinical trials.

This study had several strengths. Data were collected in a rigorous and standardised manner across multiple different geographic and epidemiologic settings in a large study with very low loss to follow up. However, there were also some important limitations. We did not collect qualitative data on parental perceptions of readmission. The study may have missed some readmissions to non-study hospitals and where reported they lacked details of diagnoses. Data on non-hospital healthcare seeking, including outpatient clinics and traditional or faith healers were not collected.^24^

In conclusion, readmission was 4-fold more common than post-discharge mortality with wide variation across sites reflecting differing patient profiles, resources, socioeconomic constraints, and the factors limiting re-presentation to hospital. Readmissions may be appropriate and necessary to reduce post-discharge mortality in high mortality settings. Targeting children based on risk should be prioritised, including recognition of broader post-discharge risks, optimising discharge planning and communication, improving post-discharge continuity of care and providing caregiver support. The ratio of readmission to post-discharge mortality rates is a potentially useful new tool for assessing overall post-discharge provision and uptake of care.

## Contributors

JAB and JLW contributed to funding. AFS, CLL, DMD, EMu, JAB, JLW, JJ, KDT, MMa, MJC, MMN, PS, RHB, SMo, SAA, TA, and WPV designed the study. AHD, AFS, BOS, CT, CAO, CB, COO, CLL, CMar, CL, DIM, DC, JAB, JN, JM, JT, JLW, KDT, MJC, MT, MMb, NN, PA, PS, RHB, RMB, SMo, WPV, and ZK coordinated the study. AHD, AFS, BOS, CB, CLL, CMan, CJM, DMD, EMb, EMu, GSM, JAB, JM, JT, JLW, KDT, MMa, MJC, MT, PS, RHB, SMo, SMw, SAA, TA, WPV, and ZK supervised the study. AMS, AFK, COO, CMan, CJM, CL, DA, DM, EMb IO, JN, JM, LS, MH, MT, PPH, PS, SMw, SNS, SMA, and SB collected the data. AHD, BSG, CAO, CB, CMar, EC, JJ, MMb, MMN, NN, PA, and RMB managed the data. AHD, CT, RMB, and ZK did the laboratory analysis. JAB, and MMN analysed the data. AHD, BSG, CAO, CB, CLL, DB, DMD, EMu, IO, JAB, JLW, KDT, MJC, MMN, PS, RHB, RMB, SMo, SAA, TA, and WPV interpreted the data. JAB, WPV, AMS, and MMN wrote the first draft. AHD, AMS, AFK, AFS, BOS, BSG, CT, CAO, CB, COO, CLL, CMan, CJM, CMar, CL, DB, DA, DM, DMD, DIM, DC, EMb, EMu, GSM, IO, JAB, JN, JM, JT, JDC, JLW, JJ, KDT, LS, MMa, MH, MJC, MT, MMb, MMN, NN, PA, PPH, PS, RHB, RMB, SMo, SMw, SNS, SAA, SMA, SB, TA, WPV, and ZK critically reviewed the manuscript. MMN, NN and JAB had full access to the study. All authors read and approved the final version of the manuscript. JAB and JLW had final responsibility for the decision to submit for publication.

## Data sharing statement

The CHAIN cohort data and analysis code are deposited and may be requested at the Harvard Dataverse website https://dataverse.harvard.edu/dataverse/chain.

## Declaration of interests

Contributors received grant funding to their institutions from the Bill & Melinda Gates Foundation in relation to this study and allied work and declare no other competing interests.
