# Supplementary material for: Hospital readmission following acute illness among children 2–23 months old in sub-Saharan Africa and South Asia: a secondary analysis of CHAIN cohort
Source: eClinicalMedicine. 2024 Jun 7;73:102676. doi: 10.1016/j.eclinm.2024.102676 (PMC11200276; doi:10.1016/j.eclinm.2024.102676)
Supplement: Supplementary Appendix [file mmc1.docx]

**Hospital readmission following acute illness among children 2‒23 months old in sub-Saharan Africa and South Asia: a secondary analysis of CHAIN cohort.**

**The Childhood Acute Illness and Nutrition (CHAIN) Network***

**Supplementary appendix**

Table of Contents

[**The CHAIN Network Collaborators** 4](#_Toc166842284)

[**Supplementary Methods** 7](#_Toc166842285)

[**Clinical variables** 7](#_Toc166842286)

[**Household data** 8](#_Toc166842287)

[**Caregiver data** 9](#_Toc166842288)

[**Access to healthcare data** 9](#_Toc166842289)

[**Domains** 9](#_Toc166842290)

[**Supplementary results** 11](#_Toc166842291)

[Supplementary Table 1. Child domain scores at index admission and discharge. 11](#_Toc166842292)

[Supplementary Table 2. Episodes of hospital readmission stratified by cohort strata. 12](#_Toc166842293)

[Supplementary Table 3. Monthly hospital readmission rates. 13](#_Toc166842294)

[Supplementary Table 4. Readmission rates by index admission syndrome. 13](#_Toc166842295)

[Supplementary Table 5. Hospital readmission and post-discharge mortality rates by site. 15](#_Toc166842296)

[Supplementary Table 6. Hospital readmission and post-discharge mortality rates by age group. 15](#_Toc166842297)

[Supplementary Table 7. Child characteristics at the time of readmission among children readmitted at the study hospitals. 16](#_Toc166842298)

[Supplementary Table 8. Most frequent diagnoses assigned by clinician following condition-specific index admission. 19](#_Toc166842299)

[Supplementary Table 9. Hospital readmission and post-discharge mortality rates ratios by cohort strata. 19](#_Toc166842300)

[Supplementary Table 10. Univariate analysis of characteristics associated with hospital readmission. 21](#_Toc166842301)

[Supplementary Table 11. Multivariable regression models with individual variables. 22](#_Toc166842302)

[**Supplementary Figures** 27](#_Toc166842303)

[Supplementary Figure 1. Flow of participants. 27](#_Toc166842304)

[Supplementary Figure 2. Cumulative hazard of readmission by admission clinical syndromes/diagnosis of: a) Chronic conditions, b) HIV, and c) Severe Pneumonia. 29](#_Toc166842305)

[Supplementary Figure 3. Cumulative hazard of readmission by site. 30](#_Toc166842306)

[Supplementary Figure 5. Linear regression of non-fatal hospital readmission and post-discharge death rate across selected groups. 32](#_Toc166842307)

[Supplementary Figure 6. Scatter plot of hospital readmissions (fatal and non-fatal) and post-discharge mortality rates across the sites. 33](#_Toc166842308)

[34](#_Toc166842309)

[**Statistical Analysis Plan** 34](#_Toc166842310)

[*1.* *Background* 37](#_Toc166842311)

[*2.* *Objectives* 37](#_Toc166842312)

[*2.1.* *General objective* 37](#_Toc166842313)

[*3.* *Study Design* 38](#_Toc166842314)

[*3.1.* *Setting* 38](#_Toc166842315)

[*3.2.* *Participants* 38](#_Toc166842316)

[*Inclusion criteria* 38](#_Toc166842317)

[*Exclusion criteria* 39](#_Toc166842318)

[*Stratification* 39](#_Toc166842319)

[*Timelines* 39](#_Toc166842320)

[*3.3.* *Variables* 40](#_Toc166842321)

[*Outcomes* 40](#_Toc166842322)

[*Exposures* 40](#_Toc166842323)

[*Potential confounders and effect modifiers* 41](#_Toc166842324)

[*3.4.* *Bias* 41](#_Toc166842325)

[*3.5.* *Study Size* 42](#_Toc166842326)

[*3.6.* *Quantitative variables* 43](#_Toc166842327)

[*4.* *Statistical methods* 44](#_Toc166842328)

[*4.1.* *Participants* 44](#_Toc166842329)

[*5.* *Baseline characteristics* 44](#_Toc166842330)

[*6.* *Follow up* 44](#_Toc166842331)

[*7.* *Outcome data* 44](#_Toc166842332)

[*7.1.* *Main results* 44](#_Toc166842333)

[*7.2.* *Summary of analyses* 45](#_Toc166842334)

[*7.3.* *Regression models - methods* 46](#_Toc166842335)

[*Binomial regression models* 46](#_Toc166842336)

[*Survival regression models* 46](#_Toc166842337)

[*5.0* *Prespecified sub analyses* 47](#_Toc166842338)

[*References* 48](#_Toc166842339)

# **The CHAIN Network Collaborators**

**Contributors’ full names and academic degrees listed alphabetically**

Professor Abdoulaye Hama Diallo, M.D., Ph.D.^1,2^, Abu Sadat Mohammad Sayeem Bin Shahid, M.B.B.S.^3^, Al Fazal Khan, M.B.B.S., Ph.D.^4^, Ali Faisal Saleem, P.G.Dip.^5^, M.Sc., M.B.B.S., F.C.P.S., Benson O. Singa, M.P.H., M.B.Ch.B^6^, Blaise Siézan Gnoumou, M.Sc.^2^, Caroline Tigoi, M.Sc.^7,8^, Catherine Achieng^6^, Celine Bourdon, M.Sc.^9^, Chris Oduol^6^, Christina L. Lancioni^10^, M.D., Christine Manyasi, M.Med.^11^, Christine J. McGrath, M.P.H.^12^, Ph.D., Christopher Maronga, M.Sc.^7^, Christopher Lwanga, M.B.Ch.B.^14^, Daniella Brals Ph.D.^15^, Dilruba Ahmed, M.B.B.S., Ph.D.^16^, Dinesh Mondal, M.B.B.S., M.D., Ph.D.^3^, Professor Donna M. Denno, M.P.H., M.D.^17^, Dorothy I. Mangale, M.P.H.^12^, Emmanuel Chimezi, B.Sc.^18^, Emmie Mbale, M.Sc., M.B.B.S.^18^, Ezekiel Mupere, M.B.Ch.B., Ph.D.^19^, Gazi Md. Salauddin Mamun, M.P.H., M.B.B.S.^20^, Issaka Ouédraogo, M.Med., M.D.^21^, Professor James A. Berkley, F.R.C.P.CH., F.Med.Sci.^7,8^, Jenala Njirammadzi, M.B.B.S.^18^, John Mukisa, M.Sc., M.B.Ch.B^22^, Johnstone Thitiri, M.Sc.^7^, Professor Judd L. Walson, M.P.H., M.D.^23^, Julie Jemutai, Ph.D.^7^, Kirkby D. Tickell, M.P.H., M.B.B.S., Ph.D.^12^, Lubaba Shahrin, M.B.B.S., F.C.P.S.^24^, MacPherson Mallewa, M.B.B.S., Ph.D.^18^, Md. Iqbal Hossain, M.B.B.S., Ph.D.^3^, Mohammod Jobayer Chisti. M.Med., M.B.B.S., Ph.D.^3^, Molly Timbwa, B.Sc.^7^, Moses Mburu, B.Sc.^7^, Moses M. Ngari, D.Phil.^7^, Narshion Ngao, M.Sc.^7^, Peace Aber, M.Stat.^14^, Philliness Prisca Harawa, M.B.B.S.^18^, Priya Sukhtankar, M.B.Ch.B.^7^, Robert H. J. Bandsma, M.D., Ph.D.^25,26,27^, Roseline Maïmouna Bamouni, M.P.H.^1^, Professor Sassy Molyneux, Ph.D. ^28^, Shalton Mwaringa, H.N.D.^7^, Shamsun Nahar Shaima, M.P.H., M.B.B.S.^3^, Syed Asad Ali, M.P.H., F.A.A.P., M.B.B.S.^5^, Syeda Momena Afsana, M.Phil., M.B.B.S.^29^, Syera Banu, M.B.B.S., Ph.D.^20^, Professor Tahmeed Ahmed, M.B.B.S., Ph.D.^24^, Wieger P. Voskuijl, M.D., Ph.D.^30^, Zaubina Kazi, B.D.S., M.Sc.^5^

**Affiliations**:

1-Department of Public Health, University Joseph Ki-Zerbo, Ouagadougou, Burkina Faso

2-Department of Public Health, Centre Muraz Research Institute, Bobo-Dioulasso, Burkina Faso

3-Nutrition Research Division, International Centre for Diarrhoeal Disease Research, Bangladesh (icddr,b), Dhaka, Bangladesh

4-Health System and Population Studies Division, International Centre for Diarrhoeal Disease Research, Bangladesh (icddr,b), Dhaka, Bangladesh

5-Department of Pediatrics and Child Health, Aga Khan University, Karachi, Pakistan

6-Kenya Medical Research Institute, Nairobi, Kenya

7- Clinical Research Department, KEMRI–Wellcome Trust Research Programme, Kilifi, Kenya

8- Centre for Tropical Medicine and Global Health, Nuffield Department of Medicine, University of Oxford, Oxford, United Kingdom

9-Translational Medicine, Hospital for Sick Children, Toronto, ON, Canada

10-Department of Pediatrics, Oregon Health and Science University, Portland, OR, USA

11-Department of Paediatrics, Mbagathi Hospital, Nairobi, Kenya

12-Department of Global Health, University of Washington, Seattle, WA, USA

13-Department of Epidemiology, University of Washington, Seattle, WA, USA

14-Uganda-Case Western Reserve University Research Collaboration, Kampala, Uganda

15-Department of Global Health, Amsterdam UMC, University of Amsterdam, Amsterdam, Netherlands

16-Clinical Microbiology and Immunology Laboratory, Office of Executive Director, International Centre for Diarrhoeal Disease Research, Bangladesh (icddr,b), Dhaka, Bangladesh

17-Department of Pediatrics, University of Washington, Seattle, WA, USA

18-Department of Paediatrics and Child Health, Kamuzu University of Health Sciences, Blantyre, Malawi

19-Department of Paediatrics and Child Health, Makerere University College of Health Sciences, Kampala, Uganda

20-Infectious Diseases Division, International Centre for Diarrhoeal Disease Research Bangladesh (icddr,b), Dhaka, Bangladesh

21-Department of Pediatrics, Banfora Referral Regional Hospital, Banfora, Burkina Faso

22-Department of Immunology and Department of Molecular Biology Makerere University College of Health Sciences, Kampala, Uganda

23-Departments of International Health and Medicine, Bloomberg School of Public Health, Johns Hopkins University, Baltimore, MD, USA

24- Office of the Director, International Centre for Diarrhoeal Disease Research, Bangladesh (icddr,b), Dhaka, Bangladesh

25-Centre for Global Child Health, Hospital for Sick Children, Toronto, ON, Canada.

26-Department of Nutritional Sciences, Faculty of Medicine, University of Toronto, Toronto, ON, Canada.

27-Department of Biomedical Sciences, College of Medicine, University of Malawi, Blantyre, Malawi.

28-Health Systems and Research Ethics Department, KEMRI–Wellcome Trust Research Programme, Kilifi, Kenya.

29-Clinical Biochemistry Laboratory, Office of Executive Director, International Centre for Diarrhoeal Disease Research, Bangladesh (icddr,b), Dhaka, Bangladesh.

30- Amsterdam University Medical Centre, Amsterdam Institute for Global Child Health, Emma Children's Hospital, Meibergdreef 9, 1105 AZ, Amsterdam, The Netherlands.

**Contributors’ roles**

JAB and JLW obtained funding. AFS, CLL, DMD, EMu, JAB, JLW, JJ, KDT, MMa, MJC, MMN, PS, RHB, SMo, SAA, TA, and WPV designed the study. AHD, AFS, BOS, CT, CAO, CB, COO, CLL, CMar, CL, DIM, DC, JAB, JN, JM, JT, JLW, KDT, MJC, MT, MMb, NN, PA, PS, RHB, RMB, SMo, WPV, and ZK coordinated the study. AHD, AFS, BOS, CB, CLL, CMan, CJM, DMD, EMb, EMu, GSM, JAB, JM, JT, JLW, KDT, MMa, MJC, MT, PS, RHB, SMo, SMw, SAA, TA, WPV, and ZK supervised the study. AMS, AFK, COO, CMan, CJM, CL, DA, DM, EMb IO, JN, JM, LS, MH, MT, PPH, PS, SMw, SNS, SMA, and SB collected the data. AHD, BSG, CAO, CB, CMar, EC, JJ, MMb, MMN, NN, PA, and RMB managed the data. AHD, CT, RMB, and ZK did the laboratory analysis. JAB, and MMN analysed the data. AHD, BSG, CAO, CB, CLL, DB, DMD, EMu, IO, JAB, JLW, KDT, MJC, MMN, PS, RHB, RMB, SMo, SAA, TA, and WPV interpreted the data. JAB, WPV, AMS, and MMN wrote the first draft. AHD, AMS, AFK, AFS, BOS, BSG, CT, CAO, CB, COO, CLL, CMan, CJM, CMar, CL, DB, DA, DM, DMD, DIM, DC, EMb, EMu, GSM, IO, JAB, JN, JM, JT, JDC, JLW, JJ, KDT, LS, MMa, MH, MJC, MT, MMb, MMN, NN, PA, PPH, PS, RHB, RMB, SMo, SMw, SNS, SAA, SMA, SB, TA, WPV, and ZK critically reviewed the manuscript. MMN verified the underlying data reported in the manuscript. All members of the writing group had full access to all the data in the study and had final responsibility for the decision to submit for publication.

**Contributors’ affiliations**

Amsterdam Centre for Global Child Health, Emma Children's Hospital; Amsterdam University Medical Centres, Amsterdam (DB, WPV); Centre for Global Child Health (RHJB), Translational Medicine (CB), Hospital for Sick Children, Toronto; Centre for Tropical Medicine & Global Health, Nuffield Department of Medicine, University of Oxford (JAB., PS, SMo); Clinical Research Department, KEMRI-Wellcome Trust Research Programme, Kilifi, Kenya (CM, CT, JAB, JJ, JT, MM, MN, MT, NN, PS, SMw); Health Systems and Research Ethics Department, KEMRI-Wellcome Trust Research Programme, Kilifi, Kenya (SMo); Departments of Epidemiology (JLW, KDT), Global Health (CJM, DIM, DMD, JDC, JLW, KDT), Medicine (JLW), Pediatrics (DMD, JLW), University of Washington, Seattle; Departments of Immunology and Molecular Biology (JM), Paediatrics and Child Health (EM), Makerere University College of Health Sciences, Kampala; Department of Nutritional Sciences, Faculty of Medicine, University of Toronto, Toronto (RHJB); Department of Paediatrics, Mbagathi Hospital, Nairobi (CM); Department of Pediatrics, Banfora Referral Regional Hospital, Banfora (IO); Department of International Health, Bloomberg School of Public Health, Johns Hopkins University (JLW); Department of Pediatrics, Oregon Health & Science University, Portland (CLL) ; Department of Pediatrics and Child Health, Aga Khan University, Karachi (AFS, SAA, ZK); Departments of Paediatrics and Child Health (EC, EM, JN, MM, PPH), Biomedical Sciences (RHJB) Kamuzu University of Health Sciences, Blantyre, Malawi; Department of Public Health, University Joseph KI-ZERBO, Ouagadougou (AHD, BSG RMD); Department of Public Health, Centre MURAZ Research Institute, Bobo-Dioulasso (AHD); Divisions of Infectious Diseases (SB), Laboratory Sciences and Services (DA, SMA), Nutrition and Clinical Services (AFK, ASM, SBS, DM, GMSM, LS, MIH, MJC, SNS, TA), International Centre for Diarrhoeal Disease Research (icddr,b), Dhaka; Kenya Medical Research Institute, Nairobi (BOS, CA, CO); Uganda-Case Western Reserve University Research Collaboration (CL, PA).

MN, NN and JAB had full access and have verified the data.

**Contributors’ contact information**

Abdoulaye Hama Diallo, hamadial@yahoo.fr, Abu Sadat Mohammad Sayeem Bin Shahid, sayeem@icddrb.org, Al Fazal Khan, fazal@icddrb.org, Ali Faisal Saleem, ali.saleem@aku.edu, Benson O. Singa, singabo2008@gmail.com, Blaise Siézan Gnoumou, blaise.gnoumou@gmail.com, Caroline Tigoi, ctigoi@kemri-wellcome.org, Catherine Achieng, catheotieno@gmail.com, Celine Bourdon, celine.bourdon@sickkids.ca, Chris Oduol, oduolchris@gmail.com, Christina L. Lancioni, lancioni@ohsu.edu, Christine Manyasi, nmanyasi@yahoo.com, Christine J. McGrath, mcgrathc@uw.edu, Christopher Maronga, marongachristopher@gmail.com, Christopher Lwanga, christopherlwanga1@gmail.com, Daniella Brals d.brals@aighd.org, Dilruba Ahmed, dahmed@icddrb.org, Dinesh Mondal, din63d@icddrb.org, Donna M. Denno, ddenno@uw.edu, Dorothy I. Mangale, dmangale@uw.edu, Emmanuel Chimezi, echimwezi@medcol.mw, Emmie Mbale, emmiembale@gmail.com, Ezekiel Mupere, mupez@yahoo.com, Gazi Md. Salauddin Mamun, gazi.mamun@icddrb.org, Issaka Ouédraogo, osbareoued@yahoo.com, James A. Berkley, jberkley@kemri-wellcome.org, Jenala Njirammadzi, jnjirammadzi@medcol.mw, John Mukisa, jmukisa90@gmail.com, Johnstone Thitiri, jthitiri@kemri-wellcome.org, Judd L. Walson, jwalson1@jhu.edu, Julie Jemutai, jemutai.julie@outlook.com, Kirkby D. Tickell, kirkbt@uw.edu, Lubaba Shahrin, lubabashahrin@icddrb.org, MacPherson Mallewa, mmallewa@medcol.mw, Md. Iqbal Hossain, ihossain@icddrb.org, Mohammod Jobayer Chisti, chisti@icddrb.org, Molly Timbwa, mtimbwa@kemri-wellcome.org, Moses Mburu, moses.mburu@finddx.org, Moses M. Ngari, mngari@kemri-wellcome.org, Narshion Ngao, nngao@kemri-wellcome.org, Peace Aber, peaceaber@gmail.com, Philliness Prisca Harawa, ppharawa@medcol.mw, Priya Sukhtankar, psukhtankar@kemri-wellcome.org, Robert H. J. Bandsma, robert.bandsma@sickkids.ca, Roseline Maïmouna Bamouni, m.bamouni@hotmail.com, Sassy Molyneux smolyneux@kemri-wellcome.org, Shalton Mwaringa, smwaringa@kemri-wellcome.org, Shamsun Nahar Shaima, shamsun.shaima@icddrb.org, Syed Asad Ali, asad.ali@aku.edu, Syeda Momena Afsana, momena@icddrb.org, Syera Banu, sbanu@icddrb.org, Tahmeed Ahmed, tahmeed@icddrb.org, Wieger P. Voskuijl, w.p.voskuijl@amsterdamumc.nl, Zaubina Kazi, zaubina.kazi@aku.edu.

# **Supplementary Methods**

## **Clinical variables**

All clinical variables definition and diagnosis followed the World Health Organization (WHO) or international guidelines as explained below;

- Anaemia – haemoglobin was categorized into four groups following WHO guidelines: none (haemoglobin >11g/dl), mild (haemoglobin ≥10 to 11g/dl), moderate (haemoglobin ≥7 to <10g/dl) and severe (haemoglobin <7g/dl).
- Blood glucose ‒ reported in two groups: normal (blood glucose 3 to 10 mmol/L) and abnormal (blood glucose <3mmol/L or >10mmol/L).
- Born small for gestational age (SGA); children reported birth weight <2.5kg or born premature (gestational age <37 weeks).
- Dehydration – categorized into three groups; none (none of the Integrated Management of Childhood Illness signs), some dehydration (two of: restless/irritable, sunken eyes, drinks eagerly/thirsty, skin pinch goes back slowly) and severe dehydration (lethargic/unconscious, sunken eyes, not able to drink or drinking poorly, skin pinch goes back very slowly).
- Diarrhoea – passage of three or more loose or watery stools in a 24 hrs period.
- Malaria – positive rapid Malaria test.
- Severe pneumonia – defined as presence of cough and/or difficulty breathing with either any of the following signs central cyanosis, oxygen saturation <90%, lower chest wall indrawing, inability to drink/breast fed/vomiting everything and impaired consciousness.
- Systemic Inflammatory Response Syndrome (SIRS) – defined as presence of at least two of the following four criteria; heart rate low (<90) or high (>180)/min; temperature low (<36°C) or high (≥38.5°C); respiratory rate high (>34 breaths per minute) and WBC low (<5 x10^9^/l) or high (>17.5 x10^9^/l).
- Tuberculosis – any child put on TB treatment.
- Chronic conditions included diagnosis of thalassemia, known TB, cerebral palsy, sickle cell disease or congenital cardiac disease at admission or index discharge.

## **Household data**

Household food insecurity was assessed using a set of eight questions adapted from Food Insecurity Experience Scale (FIES). All the questions were added (yes=1 and no=0) and the total score categorised into three level: low (0 to 3), moderate (4 to 6) and severe (7 to 8) food insecurity, respectively.

Water hygiene and sanitation (WASH) facilities present in the household were categorised into improved and unimproved. Improved water sources included piped water in the household, public tab, borehole, protected dug well, protected spring, rainwater collection and bottled water. Unimproved water sources were unprotected dug well, unprotected spring, vendor-provided water, tanker truck water and surface water (river, stream, dam, lake, pond, canal, irrigation channel).

Principal component analysis (PCA) was used to calculate household asset index by using assets and housing structure adapted from the Demographic and Health Survey (KDHS). Assets and housing structure missing data were imputed using the iterative PCA method before running PCA on complete observations. Household assets indices were developed separately for rural and urban settings. The household asset index was expressed in terms of quintiles with five categories depicting from the poorest to the least poor with each category representing approximately 20% of the participants.

Child dietary diversity score was calculated as the sum of food consumed in the last week from the seven food groups (dairy products, eggs, flesh foods, vegetables, grains, legumes & nuts and roots & tuners) plus currently breast feeding. Children <6 months old who were exclusively breastfed, aged 6 to 9 months had breastmilk plus at least two food types and those aged ≥10 months had breastmilk and at least four food types were considered to have the recommended adequate diet.

## **Caregiver data**

Caregiver’s data were collected within 48 hours of admission. Demographics (age, level of education), anthropometric measurements (Body Mass Index) and whether she was sick at the time of the interview were collected. The Patient Health Questionnaire 9-item (PHQ-9) tool with an additional tenth question on overall functional impairment was used to assess caregiver’s mental health. Total scores from the PHQ-9 questions were calculated and categorised into six groups: as none (0), minimal (1 to 4), mild (5 to 9), moderate (10 to 14), moderately severe (15 to 20) and severe (≥20).

## **Access to healthcare data**

After index hospital discharge, children were escorted home by study team where GPS coordinates of a participant was taken. Using the household GPS coordinates, euclidian distances to the nearest health facilities and study hospital were estimated. Data on travel cost to the study hospital (converted to US dollars) and means of travel were also collected.

## **Domains**

Individual data variables were grouped into preconceived domains that were used in the regression modelling. Exploratory factor analysis using correlations and other factor loadings were used to choose variables that best fitted a domain where theoretically variables could be in different domains. Within each domain, confirmatory factor analysis was conducted to estimate the latent variable domain score which were categorised into tertiles for analysis. In brief there were seven domains: underlying medical conditions, child-level nutritional risk exposures, signs of illness severity at admission, signs of illness severity at discharge, access to health care, household-level exposures and caregiver characteristics. Individual variables in each domain are shown below.

| **The domain** | **Variables within domains** |
| --- | --- |
| **Underlying medical conditions** |  |
|  | Small size at birth size (no, yes) |
|  | Height-for-age z score |
|  | Prior hospitalization (no, >1 month, <1 month) |
|  | Chronic conditions (none, suspected/confirmed) |
| **Child-level nutritional risk exposures** | Recommended appropriate diet (yes, no) |
|  | Recent weight loss (none, suspected, confirmed) |
|  | Poor feeding (no, yes) |
| **Signs of illness severity at admission** | Systemic Inflammatory Response Syndrome (no, yes) |
|  | Respiratory distress (none, moderate, severe) |
|  | Circulation (none, some signs, all signs) |
|  | Conscious level (A, VPU) |
|  | Dehydration (none, some, severe) |
|  | Blood glucose (normal, abnormal) |
|  | Severe anaemia (no, yes) |
| **Signs of illness severity at discharge** | Systemic Inflammatory Response Syndrome (no, yes) |
|  | Respiratory distress (none, moderate, severe) |
|  | Circulation (none, some signs) |
|  | Dehydration (none, some, severe) |
|  | Severe anaemia (no, yes) |
| **Access to health care** | Distance to the nearest health facility (km) |
|  | Means of travel to hospital |
|  | Travel cost |
|  | Travel time |
| **Household-level exposures** | Assets quintiles |
|  | Food insecurity (low, medium, high) |
|  | Type of toilet (improved, not improved) |
|  | Water availability (yes, no) |
| **Caregiver characteristics** | Biological mother as primary caregiver (yes, no) |
|  | Caregiver education level |
|  | Mother mental health |
|  | Mother sick (no, yes) |
|  | Mother working |

# **Supplementary results**

## Supplementary Table 1. Child domain scores at index admission and discharge.

|  | **All participants (N=2874)** | **No readmission (N=2368)** | **At least one readmission  (N=506)** | **P-value** |
| --- | --- | --- | --- | --- |
| **Signs of illness severity at admission** | | | |  |
| Low | 1059 (37) | 898 (38) | 161 (32) |  |
| Medium | 919 (32) | 760 (32) | 159 (31) | 0.005 |
| High | 896 (31) | 710 (30) | 186 (37) |  |
| **Signs of illness severity at discharge** | | | |  |
| Low | 2382 (83) | 1963 (83) | 419 (83) |  |
| Medium | 422 (15) | 344 (15) | 78 (15) | 0.52 |
| High | 70 (2.4) | 61 (2.6) | 9 (1.8) |  |
| **Underlying medical conditions** | | | |  |
| Low | 1007 (35) | 880 (37) | 127 (25) |  |
| Medium | 983 (34) | 797 (34) | 186 (37) | <0.001 |
| High | 884 (31) | 691 (29) | 193 (38) |  |
| **Child-level nutritional risk exposures** | | | |  |
| Low | 1655 (58) | 1364 (58) | 291 (58) |  |
| Medium | 306 (11) | 253 (11) | 53 (10) | 0.99 |
| High | 913 (32) | 751 (32) | 162 (32) |  |
| **Caregiver characteristics** | | | |  |
| Least adverse | 1143 (40) | 957 (40) | 186 (37) |  |
| Moderately adverse | 919 (32) | 752 (32) | 167 (33) | 0.29 |
| Most adverse | 812 (28) | 659 (28) | 153 (30) |  |
| **Household-level exposures** | | | |  |
| Least adverse | 1009 (35) | 805 (34) | 204 (40) |  |
| Moderately adverse | 950 (33) | 787 (33) | 163 (32) | 0.01 |
| Most adverse | 915 (32) | 776 (33) | 139 (27) |  |
| **Access to health care** | | | |  |
| Least adverse | 952 (33) | 779 (33) | 173 (34) |  |
| Moderately adverse | 978 (34) | 795 (34) | 183 (36) | 0.23 |
| Most adverse | 944 (33) | 794 (34) | 150 (30) |  |
| The results are N (%). | | | | |

## Supplementary Table 2. Episodes of hospital readmission stratified by cohort strata.

| **Nutritional status strata**  **(N=2874)** | **N** | **All hospital readmissions** | | **Hospital readmissions within 1 month after index discharge** | | **Hospital readmissions after 1 month following index discharge** | | **Rate ratios*** | **P-value** |
| --- | --- | --- | --- | --- | --- | --- | --- | --- | --- |
|  |  | Episodes | Readmission rate (95%CI)/1000 child-months | Episodes | Readmission rate (95%CI)/1000 child-months | Episodes | Readmission rate (95%CI)/1000 child-months | Comparing early readmission with late readmission rates** |  |
| NW | 1072 | 228 | 37.4 (32.8‒42.6) | 71 | 66.9 (53.0‒84.4) | 157 | 29.7 (25.4‒34.7) | 2.05 (1.54‒2.73) | <0.001 |
| MW | 724 | 159 | 38.9 (33.3‒45.4) | 50 | 70.0 (51.8‒90.7) | 109 | 30.6 (25.3‒36.9) | 1.94 (1.39‒2.71) | 0.0001 |
| SWK | 1078 | 268 | 46.4 (41.1‒52.3) | 92 | 88.3 (71.9‒108) | 176 | 35.7 (30.8‒41.3) | 1.88 (1.45‒2.45) | <0.001 |
| All children | 2874 | 655 | 41.0 (38.0‒44.3) | 213 | 75.6 (66.1‒86.4) | 442 | 32.0 (29.2‒35.2) | 2.01 (1.70‒2.37) | <0.001 |

*Adjusted for age, sex and recruiting hospital, NW; Not wasted, MW; moderate wasted, SWK; Severe wasted/Kwashiorkor.

** Ealy readmissions are within 30 days of index discharge; late readmissions are during days 31 to 180.

## Supplementary Table 3. Monthly hospital readmission rates.

| **Month** | **Not wasted (N=228)** | | **Moderate wasted (N=159)** | | **Severe wasted/Kwashiorkor (N=268)** | | **Overall (N=655)** | |
| --- | --- | --- | --- | --- | --- | --- | --- | --- |
|  | Episodes | Rate (95%CI) | Episodes | Rate (95%CI) | Episodes | Rate (95%CI) | Episodes | Rate (95%CI) |
| 0 to 1 | 71 | 66.9 (53.0‒84.4) | 50 | 69.9 (53.0‒92.3) | 92 | 88.3 (71.9‒108) | 213 | 75.6 (66.1‒86.4) |
| 1 to 2 | 46 | 43.7 (32.8‒58.4) | 32 | 45.4 (32.1‒64.2) | 40 | 39.8 (29.2‒54.3) | 118 | 42.7 (35.7‒51.2) |
| 2 to 3 | 32 | 30.7 (21.7‒43.5) | 22 | 31.6 (20.8‒48.0) | 48 | 48.9 (36.8‒64.8) | 102 | 37.5 (30.9‒45.5) |
| 3 to 4 | 27 | 26.3 (18.0‒38.3) | 27 | 39.3 (26.9‒57.3) | 30 | 31.0 (21.7‒44.4) | 84 | 31.3 (25.3‒38.8) |
| 4 to 5 | 32 | 31.3 (22.2‒44.3) | 13 | 19.1 (11.1‒33.0) | 26 | 27.3 (18.6‒40.1) | 71 | 26.8 (21.2‒33.8) |
| 5 to 6 | 20 | 22.3 (14.4‒34.6) | 15 | 24.6 (14.8‒40.8) | 32 | 38.5 (27.2‒54.4) | 67 | 28.7 (22.6‒36.4) |
| Total | 228 | 37.4 (32.8‒42.6) | 159 | 38.9 (33.3‒45.4) | 268 | 46.4 (41.1‒52.3) | 655 | 41.0 (38.0‒44.3) |

The hospital readmissions are per 1000 child-months.

## Supplementary Table 4. Readmission rates by index admission syndrome.

| **Index admission syndrome** | **30-day readmissions** | | **180-day readmissions** | |
| --- | --- | --- | --- | --- |
|  | Readmission episodes | Readmission rate/1000 CM | Readmission episodes | Readmission rate/1000 CM |
| Severe pneumonia | 53 | 91.6 (69.9‒119.8) | 168 | 51.5 (44.3‒59.9) |
| Diarrhoea | 118 | 76.2 (63.6‒91.3) | 331 | 37.4 (33.6‒41.6) |
| Malaria (RDT positive) | 17 | 42.2 (26.3‒67.9) | 78 | 34.0 (27.3‒42.5) |
| HIV infected/exposed | 17 | 74.9 (46.6‒120.5) | 67 | 55.3 (43.5‒70.3) |
| Severe anaemia | 26 | 66.3 (45.1‒97.4) | 85 | 38.8 (31.4‒48.0) |
| Chronic conditions | 28 | 151 (104‒219) | 82 | 79.7 (64.2‒99.0) |

CM; Child-months, SIRS (Systemic Inflammatory Response Syndrome) defined as presence of two of the following four criteria; heart rate low (<90) or high (>180)/min; temperature low (<36°C) or high (≥38.5°C); respiratory rate high (>34 breaths per minute) and WBC low (<5 x10^9^/l) or high (>17.5 x10^9^/l), Chronic conditions includes thalassemia, cerebral palsy, sickle cell disease, congenital cardiac disease and known TB.

## Supplementary Table 5. Hospital readmission and post-discharge mortality rates by site.

| **Site** | **Hospital readmissions** | | **Post-discharge deaths** | | **Readmission rate/post-discharge mortality rate ratio (95%CI)** |
| --- | --- | --- | --- | --- | --- |
|  | **Readmission episodes** | **Readmission rates (95% CI)/ 1000 child-months*** | **Number of post-discharge deaths** | **Post-discharge mortality rate (95% CI)/ 1000 child-months*** |  |
| Kilifi | 48 | 37.1 (28.0‒49.2) | 10 | 7.73 (4.16‒14.36) | 4.80 (3.43‒6.73) |
| Mbagathi | 66 | 47.2 (37.1‒60.1) | 22 | 15.72 (10.35‒23.87) | 3.00 (2.52‒3.58) |
| Migori | 27 | 21.6 (14.8‒31.4) | 26 | 20.68 (14.08‒30.37) | 1.04 (1.03‒1.05) |
| Kampala | 104 | 41.3 (34.1‒50.1) | 24 | 9.52 (6.38‒14.20) | 4.34 (3.53‒5.34) |
| Blantyre | 92 | 64.9 (52.9‒79.7) | 18 | 12.65 (7.97‒20.07) | 5.13 (3.97‒6.64) |
| Karachi | 89 | 48.2 (39.2‒59.3) | 30 | 16.23 (11.35‒23.21) | 2.97 (2.55‒3.45) |
| Dhaka | 128 | 57.4 (48.2‒68.2) | 12 | 5.37 (3.05‒9.46) | 10.7 (7.21‒15.8) |
| Matlab | 45 | 24.8 (18.5‒33.3) | 4 | 2.20 (0.83‒5.87) | 11.3 (5.67‒22.3) |
| Banfora | 56 | 25.4 (19.6‒33.0) | 22 | 9.97 (6.57‒15.15) | 2.55 (2.19‒2.98) |

*Readmission rate test of heterogeneity P-value <0.001, post-discharge mortality rate test of heterogeneity P-value <0.001

## Supplementary Table 6. Hospital readmission and post-discharge mortality rates by age group.

| **Age (months)** | **Hospital readmissions** | | **Post-discharge deaths** | | **Readmission rate/post-discharge mortality rate ratio (95%CI)** |
| --- | --- | --- | --- | --- | --- |
|  | **Readmission episodes** | **Readmission rates (95% CI)/ 1000 child-months*** | **Number of post-discharge deaths** | **Post-discharge mortality rate (95% CI)/ 1000 child-months*** |  |
| <6 | 150 | 48.2 (41.1‒56.6) | 48 | 15.4 (11.6‒20.4) | 3.13 (2.77‒3.54) |
| 6 to 11 | 260 | 44.0 (38.9‒49.6) | 63 | 10.6 (8.31‒13.6) | 4.15 (3.65‒4.68) |
| ≥ 12 | 245 | 35.3 (31.1‒40.0) | 57 | 8.19 (6.32‒10.6) | 4.31 (3.77‒4.92) |
| Overall | 655 | 41.0 (38.0‒44.3) | 168 | 10.5 (9.03‒12.2) | 3.84 (3.06‒4.63)* |

*Readmission rate test of heterogeneity P-value <0.001, post-discharge mortality rate test of heterogeneity P-value <0.001, the I^2^ was 87.2%, test of heterogeneity of the ratios P-value <0.001, *pooled the ratio using random-effect method.

## Supplementary Table 7. Child characteristics at the time of readmission among children readmitted at the study hospitals.

|  | **Cohort strata** | | | **All readmissions (N=460)** |
| --- | --- | --- | --- | --- |
| **Initial observations** | **NW (N=148)** | **MW (N=107)** | **SW/K (N=205)** |  |
| Axillary temperature °C |  |  |  |  |
| <36 °C | 6 (4.1) | 6 (5.6) | 14 (6.8) | 26 (5.7) |
| 36 to 37.5 °C | 84 (57) | 45 (42) | 105 (51) | 234 (51) |
| >37.5 °C | 58 (39) | 56 (52) | 83 (40) | 197 (43) |
| Heart rate /minute |  |  |  |  |
| Bradycardia | 5 (3.4) | 5 (4.7) | 9 (4.4) | 19 (4.1) |
| Normal | 85 (57) | 63 (59) | 140 (68) | 288 (63) |
| Tachycardia | 58 (39) | 39 (36) | 56 (27) | 153 (33) |
| Hypoxia (Oxygen saturation (SaO2 < 90%)) | 7 (4.7) | 12 (11) | 15 (7.3) | 34 (7.4) |
| Respiratory rate /minute |  |  |  |  |
| Normal | 87 (59) | 49 (46) | 108 (53) | 244 (53) |
| Tachypnoea | 61 (41) | 58 (54) | 97 (47) | 216 (47) |
| History of previous hospitalization |  |  |  |  |
| None | 20 (14) | 13 (12) | 22 (11) | 55 (12) |
| < 1 week | 24 (16) | 14 (13) | 37 (18) | 75 (16) |
| 1 week to one month | 46 (31) | 29 (27) | 58 (28) | 133 (29) |
| >1 month | 54 (36) | 51 (48) | 82 (40) | 187 (41) |
| Anthropometry and nutrition |  |  |  |  |
| Not wasted | 61 (41) | 4 (3.7) | 6 (2.9) | 71 (15) |
| MAM | 79 (53) | 76 (71) | 54 (26) | 209 (45) |
| SAM | 5 (3.4) | 25 (23) | 134 (65) | 164 (36) |
| Missing MUAC | 3 (2.0) | 2 (1.9) | 11 (5.4) | 16 (3.5) |
| Oedema | 2 (1.4) | 2 (1.9) | 30 (15) | 34 (7.4) |
| Currently in outpatient nutrition program |  |  |  |  |
| No | 140 (95) | 77 (72) | 127 (62) | 344 (75) |
| Supplementary | 0 | 9 (8.4) | 5 (2.4) | 14 (3.0) |
| Therapeutic | 5 (3.4) | 18 (17) | 69 (34) | 92 (20) |
| Unknown | 3 (2.0) | 3 (2.8) | 4 (2.0) | 10 (2.2) |
| Has the children eaten these nutrition products in the last 3 days |  |  |  |  |
| None | 142 (96) | 84 (79) | 138 (67) | 364 (79) |
| Supplementary | 0 | 7 (6.5) | 6 (2.9) | 13 (2.8) |
| Therapeutic | 5 (3.4) | 16 (15) | 58 (28) | 79 (17) |
| Currently breastfeeding | 115 (78) | 82 (77) | 95 (46) | 292 (63) |
| **Examination at readmission** |  |  |  |  |
| Airway |  |  |  |  |
| Clear | 145 (98) | 103 (96) | 193 (94) | 441 (96) |
| Needs active support | 1 (0.7) | 4 (3.7) | 7 (3.4) | 12 (2.6) |
| Obstructed/stridor | 2 (1.4) | 0 | 2 (1.0) | 4 (0.9) |
| Breathing |  |  |  |  |
| Normal | 75 (51) | 71 (66) | 117 (57) | 263 (57) |
| Central cyanosis | 0 | 0 | 1 (0.5) | 1 (0.2) |
| Nasal flaring | 19 (13) | 25 (23) | 20 (9.8) | 64 (14) |
| Reduced air-entry | 1 (0.7) | 2 (1.9) | 1 (0.5) | 4 (0.9) |
| Wheeze | 27 (18) | 12 (11) | 18 (8.8) | 57 (12) |
| Acidotic breathing | 4 (2.7) | 3 (2.8) | 9 (4.4) | 16 (3.5) |
| Grunting | 8 (5.4) | 4 (3.7) | 6 (2.9) | 18 (3.9) |
| Lower chest wall indrawing | 38 (26) | 50 (47) | 73 (36) | 161 (35) |
| Crackles | 40 (27) | 50 (47) | 83 (40) | 173 (38) |
| Head nodding | 2 (1.4) | 3 (2.8) | 4 (2.0) | 9 (2.0) |
| Capillary refill |  |  |  |  |
| >3 seconds | 4 (2.7) | 3 (2.8) | 7 (3.4) | 14 (3.0) |
| 2 to 3 seconds | 25 (17) | 30 (28) | 47 (23) | 102 (22) |
| <2 seconds | 119 (80) | 74 (69) | 148 (72) | 341 (74) |
| Conscious level |  |  |  |  |
| Alert | 141 (95) | 100 (93) | 185 (90) | 426 (93) |
| Voice | 3 (2.0) | 1 (0.9) | 4 (2.0) | 8 (1.7) |
| Pain | 3 (2.0) | 6 (5.6) | 12 (5.9) | 21 (4.6) |
| Unresponsive | 1 (0.7) | 0 | 1 (0.5) | 2 (0.4) |
| Sunken eyes | 15 (10) | 12 (11) | 36 (18) | 63 (14) |
| Skin pinch |  |  |  |  |
| >2 seconds | 2 (1.4) | 2 (1.9) | 14 (6.8) | 18 (3.9) |
| <2 seconds | 13 (8.8) | 13 (12) | 37 (18) | 63 (14) |
| Immediate | 133 (90) | 92 (86) | 151 (74) | 376 (82) |
| Drinking/breastfeeding |  |  |  |  |
| Normal | 107 (72) | 67 (63) | 136 (66) | 310 (67) |
| Poorly | 29 (20) | 29 (27) | 48 (23) | 106 (23) |
| Not drinking | 6 (4.1) | 6 (5.6) | 13 (6.3) | 25 (5.4) |
| Eager/thirsty | 6 (4.1) | 4 (3.7) | 4 (2.0) | 14 (3.0) |
| **Initial treatment** |  |  |  |  |
| Admitted to |  |  |  |  |
| Ward | 127 (86) | 91 (85) | 158 (77) | 376 (82) |
| HDU | 10 (6.8) | 7 (6.5) | 15 (7.3) | 32 (7.0) |
| ICU | 9 (6.1) | 9 (8.4) | 19 (9.3) | 37 (8.0) |

MUAC; Mid upper arm circumference, MAM; moderate acute malnutrition, SAM; Severe acute malnutrition, HDU; Higher dependency unit, ICU; Intensive Care unit.

## Supplementary Table 8. Most frequent diagnoses assigned by clinician following condition-specific index admission.

| **Index admission diagnosis** | | **Hospital readmission diagnosis** | | | | |
| --- | --- | --- | --- | --- | --- | --- |
| Diagnosis | N (%) | Pneumonia | Gastroenteritis | Sepsis | Anaemia | Malaria |
| Pneumonia | 202 (44) | 72 | 30 | 18 | 13 | 6.4 |
| Gastroenteritis | 184 (40) | 64 | 42 | 21 | 12 | 7.1 |
| Sepsis | 76 (17) | 50 | 28 | 46 | 13 | 9.2 |
| Anaemia | 88 (19) | 47 | 32 | 15 | 53 | 31 |
| Malaria | 62 (13) | 45 | 32 | 15 | 34 | 50 |

## Supplementary Table 9. Hospital readmission and post-discharge mortality rates ratios by cohort strata.

| **Cohort strata**  **(N=2874)** | **N** | **Hospital readmissions** | | **Post-discharge deaths** | | **Readmission to deaths ratio (95% CI)** |
| --- | --- | --- | --- | --- | --- | --- |
|  |  | **Episodes** | **Readmission rate (95%CI)/1000 child-months** | **Deaths** | **Mortality rate (95%CI)/1000 child-months** |  |
| Not wasted | 1072 | 228 | 37.4 (32.8‒42.6) | 17 | 2.72 (1.72‒4.57) | 13.8 (9.32‒19.1) |
| Moderately wasted | 724 | 159 | 38.9 (33.3‒45.4) | 30 | 7.32 (5.16‒10.7) | 5.31 (4.24‒6.45) |
| Severely wasted or Kwashiorkor | 1078 | 268 | 46.4 (41.1‒52.3) | 121 | 21.0 (17.6‒25.3) | 2.21 (2.07‒2.34) |
| All children | 2874 | 655 | 41.0 (38.0‒44.3) | 168 | 10.5 (9.03‒12.2) |  |

The hospital readmissions and mortality rates are per 1000 child-months.

## Supplementary Table 10. Univariate analysis of characteristics associated with hospital readmission.

|  | **Readmission episodes (N=655)** | **SHR (95% CI)*** | **P-value** |
| --- | --- | --- | --- |
| Cohort strata | | | |
| Not wasted | 228 (35) | Reference |  |
| Moderately wasted | 159 (24) | 1.08 (0.88‒1.33) | 0.50 |
| Severely wasted or Kwashiorkor | 268 (41) | 1.23 (1.03‒1.47) | 0.02 |
| Age months (log) | - | 0.84 (0.74‒0.95) | 0.007 |
| Sex; female | 259 (40) | 0.87 (0.75‒1.02) | 0.09 |
| Left hospital against medical advice | 34 (5.2) | 0.77 (0.53‒1.12) | 0.20 |
| Admission duration days (log) | - | 1.17 (1.04‒1.31) | 0.01 |
| Index discharge on a weekend | 137 (21) | 1.00 (0.82‒1.21) | 0.94 |
| Change in anthropometry at discharge | | | |
| No change | 561 (86) | Reference |  |
| Improved | 67 (10) | 0.84 (0.65‒1.09) | 0.21 |
| Worsened | 27 (4.1) | 1.48 (1.00‒2.19) | 0.05 |
| **Signs of illness severity at admission** | | | |
| Low | 199 (30) | Reference |  |
| Medium | 204 (31) | 1.11 (0.91‒1.35) | 0.34 |
| High | 252 (39) | 1.36 (1.12‒1.65) | 0.002 |
| **Signs of illness severity at discharge** | | | |
| Low | 547 (84) | Reference |  |
| Medium | 99 (15) | 1.03 (0.82‒1.30) | 0.84 |
| High | 9 (1.4) | 0.79 (0.41‒1.52) | 0.50 |
| **HIV status** | | | |
| Negative | 559 (85) | Reference |  |
| Untested | 29 (4.4) | 1.86 (1.26‒2.74) | 0.002 |
| Exposed | 50 (7.6) | 1.40 (1.04‒1.89) | 0.03 |
| Infected | 17 (2.6) | 1.15 (0.71‒1.86) | 0.60 |
| **Underlying medical conditions** | | | |
| Low | 157 (24) | Reference |  |
| Medium | 239 (36) | 1.44 (1.18‒1.76) | <0.001 |
| High | 259 (40) | 1.69 (1.39‒2.07) | <0.001 |
| **Child-level nutritional risk exposures** | | | |
| Low | 382 (59) | Reference |  |
| Medium | 68 (10) | 1.04 (0.80‒1.34) | 0.80 |
| High | 205 (31) | 1.12 (0.94‒1.35) | 0.24 |
| **Caregiver characteristics** | | | |
| Least adverse | 231 (35) | Reference |  |
| Moderately adverse | 220 (34) | 1.21 (1.00‒1.48) | 0.05 |
| Most adverse | 204 (31) | 1.36 (1.11‒1.68) | 0.003 |
| **Household-level exposures** | | | |
| Least adverse | 277 (42) | Reference |  |
| Moderately adverse | 200 (31) | 0.96 (0.78‒1.17) | 0.71 |
| Most adverse | 178 (27) | 0.89 (0.71‒1.13) | 0.30 |
| **Access to health care** | | | |
| Least adverse | 236 (36) | Reference |  |
| Moderately adverse | 232 (35) | 1.04 (0.86‒1.26) | 0.74 |
| Most adverse | 187 (29) | 1.07 (0.85‒1.34) | 0.60 |
| *SHR- Sub-distribution Hazard ratios  All model results were weighted using sampling and lost to follow up weights.  SHR from Competing risk survival model with site as random effect. | | | |

## Supplementary Table 11. Multivariable regression models with individual variables.

|  | **Readmission episodes (N=655)** | **aSHR (95% CI)*** | **P-value** |
| --- | --- | --- | --- |
| **Demographics** | | |  |
| Sex; female | 259 (40) | 0.96 (0.82‒1.13) | 0.65 |
| Age in months | - | 0.85 (0.73‒0.98) | 0.02 |
| Cohort strata |  |  |  |
| Not wasted | 228 (35) | Reference |  |
| Moderately wasted | 159 (24) | 0.99 (0.79‒1.24) | 0.96 |
| Severely wasted/Kwashiorkor | 268 (41) | 1.04 (0.81‒1.35) | 073 |
| **Characteristics at index discharge** | | |  |
| Left hospital against medical advice | 34 (5.2) | 0.67 (0.45‒1.01) | 0.05 |
| Admission duration days | - | 1.07 (0.93‒1.23) | 0.34 |
| Index discharge on a weekend | 137 (21) | 1.06 (0.88‒1.29) | 0.57 |
| Change in anthropometry at discharge |  |  |  |
| No change | 561 (86) | Reference |  |
| Improved | 67 (10) | 0.82 (0.63‒1.09) | 0.24 |
| Worsened | 27 (4.1) | 1.48 (0.97‒2.24) | 0.07 |
| HIV status |  |  |  |
| Negative | 559 (85) | Reference |  |
| Untested | 29 (4.4) | 1.61 (1.08‒2.42) | 0.02 |
| Exposed | 50 (7.6) | 1.35 (0.99‒1.86) | 0.06 |
| Infected | 17 (2.6) | 0.96 (0.59‒1.58) | 0.96 |
| **Domain scores variables** | | |  |
| **Underlying medical conditions** |  |  |  |
| Small size at birth size (yes) | 118 (18) | 1.00 (0.80‒1.23) | 0.94 |
| Stunting |  |  |  |
| HAZ≥-2 | 285 (44) | Reference |  |
| HAZ -2 to -3 | 130 (20) | 0.96 (0.77‒1.20) | 0.75 |
| HAZ<-3 | 240 (37) | 1.15 (0.92‒1.43) | 0.25 |
| Prior hospitalization (Yes) | 248 (138) | 1.44 (1.22‒1.71) | <0.001 |
| Chronic conditions (Yes) | 82 (13) | 1.78 (1.36‒2.33) | <0.001 |
| **Child-level nutritional risk exposures** |  |  |  |
| Recommended appropriate diet (No) | 343 (52) | 1.02 (0.86‒1.21) | 0.89 |
| Recent weight loss (suspected/confirmed) | 218 (33) | 1.06 (0.76‒1.46) | 0.76 |
| Poor feeding (yes) | 118 (18) | 1.11 (0.89‒1.38) | 0.43 |
| **Signs of illness severity at admission** |  |  |  |
| SIRS (Yes) | 268 (41) | 1.21 (1.03‒1.44) | 0.03 |
| Respiratory distress |  |  |  |
| None | 325 (50) | Reference |  |
| Moderate | 130 (20) | 1.00 (0.80‒1.24) | 0.95 |
| Severe | 200 (30) | 1.24 (1.00‒1.54) | 0.05 |
| Circulation signs |  |  |  |
| None | 377 (58) | Reference |  |
| Some signs | 278 (42) | 1.10 (0.92‒1.31) | 0.34 |
| Conscious level (VPU) | 18 (2.8) | 0.84 (0.50‒1.41) | 0.57 |
| Dehydration |  |  |  |
| None | 350 (53) | Reference |  |
| Some | 269 (41) | 1.02 (0.86‒1.22) | 0.84 |
| Severe | 36 (5.5) | 0.73 (0.50‒1.06) | 0.10 |
| Blood glucose (normal, abnormal) |  |  |  |
| Normal | 609 (93) | Reference |  |
| Abnormal | 46 (7.0) | 1.01 (0.73‒1.38) | 0.95 |
| Severe anemia (yes) | 85 (13) | 1.09 (0.81‒1.47) | 0.65 |
| **Signs of illness severity at discharge** |  |  |  |
| SIRS (yes) | 104 (16) | 1.18 (0.94‒1.47) | 0.24 |
| Respiratory distress |  |  |  |
| None | 520 (79) | Reference |  |
| Moderate | 85 (13) | 1.01 (0.79‒1.30) | 0.94 |
| Severe | 50 (7.6) | 0.87 (0.62‒1.22) | 0.44 |
| Circulation |  |  |  |
| None | 563 (86) | Reference |  |
| Some signs | 92 (14) | 0.91 (0.71‒1.16) | 0.46 |
| Dehydration |  |  |  |
| None | 641 (98) | Reference |  |
| Some | 14 (2.1) | 1.27 (0.74‒2.16) | 0.45 |
| Severe anemia (yes) | 47 (7.2) | 1.14 (0.79‒1.65) | 0.54 |
| **Access to health care** |  |  |  |
| Distance to the nearest health facility (KM) | - | 0.99 (0.90‒1.08) | 0.84 |
| Means of travel to hospital |  |  |  |
| Bus/Car/Train/Ambulance | 335 (51) | Reference |  |
| Walking/Motorbike/Rickshaw/Tuktuk | 320 (49) | 0.84 (0.68‒1.04) | 0.11 |
| Travel cost |  |  |  |
| <1 US dollar | 253 (39) | 0.85 (0.60‒1.19) | 0.34 |
| 1 to <5 US dollar | 352 (54) | 1.03 (0.74‒1.42) | 0.91 |
| ≥ 5 US dollar | 50 (7.6) | Reference |  |
| Travel time |  |  |  |
| <1 hour | 312 (48) | 1.09 (0.86‒1.40) | 0.57 |
| 1 to 2 hours | 209 (32) | 0.83 (0.66‒1.04) | 0.11 |
| 2 to 4 hours | 134 (20) | Reference |  |
| Population density (People per KM^2^) |  |  |  |
| <300 | 96 (15) | Reference |  |
| 300 to 1500 | 88 (13) | 0.84 (0.59‒1.20) | 0.35 |
| >1500 | 471 (72) | 0.85 (0.58‒1.25) | 0.48 |
| **Household-level exposures** |  |  |  |
| Assets quintiles |  |  |  |
| Least poor | 160 (24) | Reference |  |
| Fourth | 169 (26) | 1.10 (0.86‒1.40) | 0.45 |
| Middle | 121 (18) | 0.97 (0.73‒1.29) | 0.96 |
| Second | 102 (16) | 1.11 (0.81‒1.53) | 0.56 |
| Poorest | 103 (16) | 1.23 (0.85‒1.80) | 0.34 |
| Food insecurity (low, medium, high) |  |  |  |
| Low | 356 (54) | Reference |  |
| Medium | 183 (28) | 0.95 (0.78‒1.16) | 0.65 |
| High | 116 (18) | 1.20 (0.94‒1.53) | 0.24 |
| Type of toilet (not improved) | 125 (19) | 0.85 (0.67‒1.08) | 0.23 |
| Water availability (not improved) | 414 (63) | 0.92 (0.77‒1.10) | 0.45 |
| **Caregiver characteristics** |  |  |  |
| Biological mother as primary caregiver (No) | 39 (6.0) | 1.08 (0.77‒1.52) | 0.76 |
| Caregiver education level |  |  |  |
| Tertiary/secondary | 229 (35) | Reference |  |
| Primary | 260 (40) | 0.83 (0.69‒1.01) | 0.07 |
| None | 166 (25) | 0.92 (0.71‒1.20) | 0.57 |
| Mother mental health |  |  |  |
| None to mild | 498 (76) | Reference |  |
| Moderate to severe | 157 (24) | 1.29 (1.06‒1.58) | 0.01 |
| Mother sick (No) | 562 (86) | 1.20 (0.95‒1.53) | 0.13 |
| Mother working |  |  |  |
| Employed | 76 (12) | Reference |  |
| No income | 442 (67) | 0.88 (0.67‒1.14) | 0.37 |
| Self-employed | 137 (21) | 1.09 (0.81‒1.46) | 0.67 |
| Change in nutritional status at discharge was defined as no change (no change in the NW, MW, SWK groups), improved (moved from SWK to MW/NW or from MW to NW) and worsened (moved from NW to MW/SWK or from MW to SWK.  Left hospital against medical advice was defined as leaving hospital against medical advice or absconding.  Small size at birth size was defined as reported birth weight <2.5kg or born premature (before 37 weeks of gestation)  HAZ-Length for age z-score  Chronic conditions included diagnosis of thalassemia, known TB, cerebral palsy, sickle cell disease or congenital cardiac disease at admission or discharge.  SIRS-systemic Inflammatory Response Syndrome | | | |

# **Supplementary Figures**


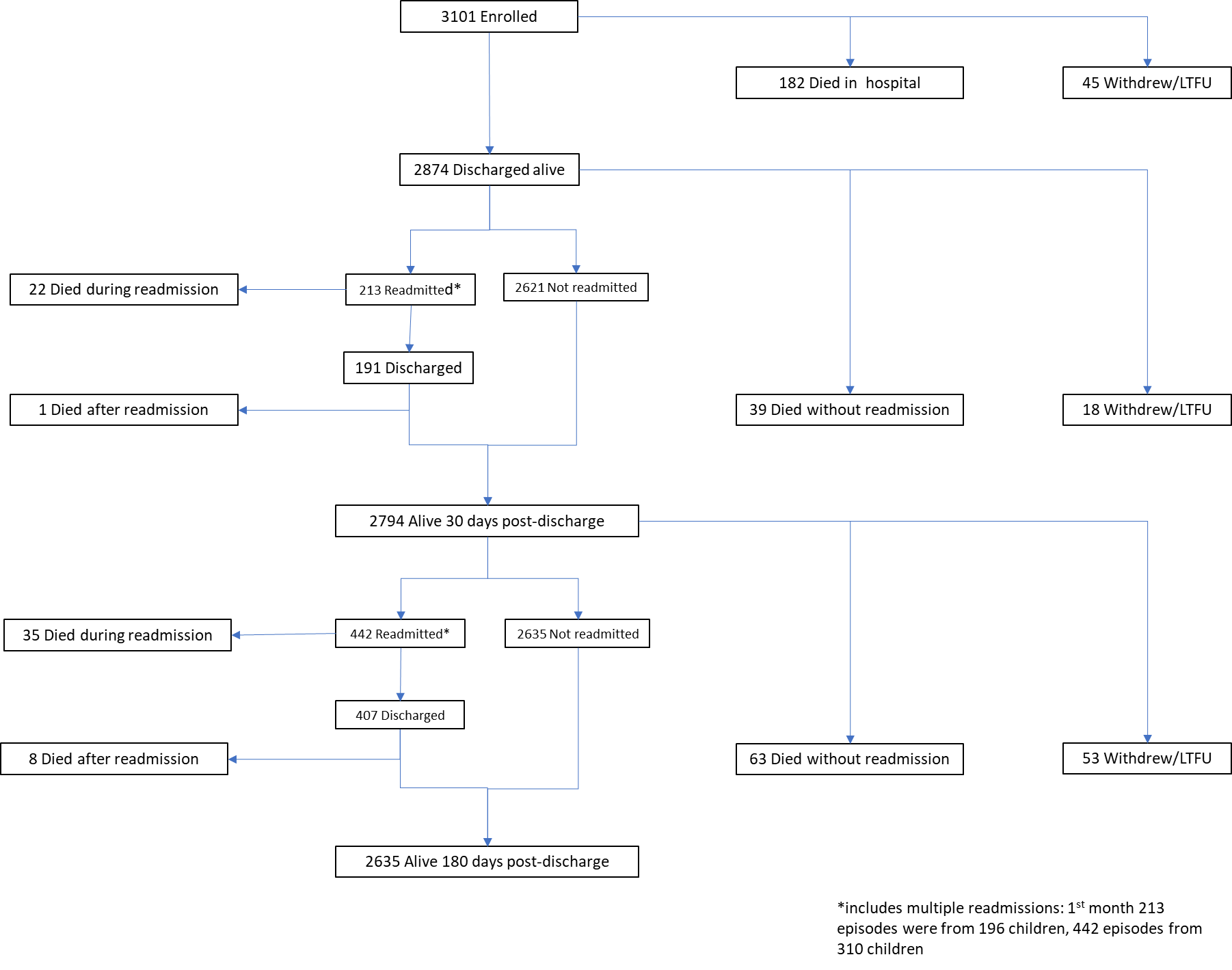


## Supplementary Figure 1. Flow of participants.

| A  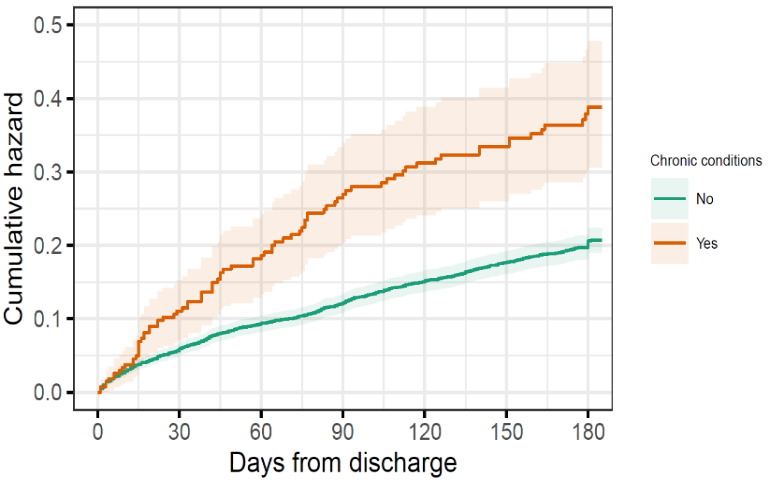 |
| --- |
| B  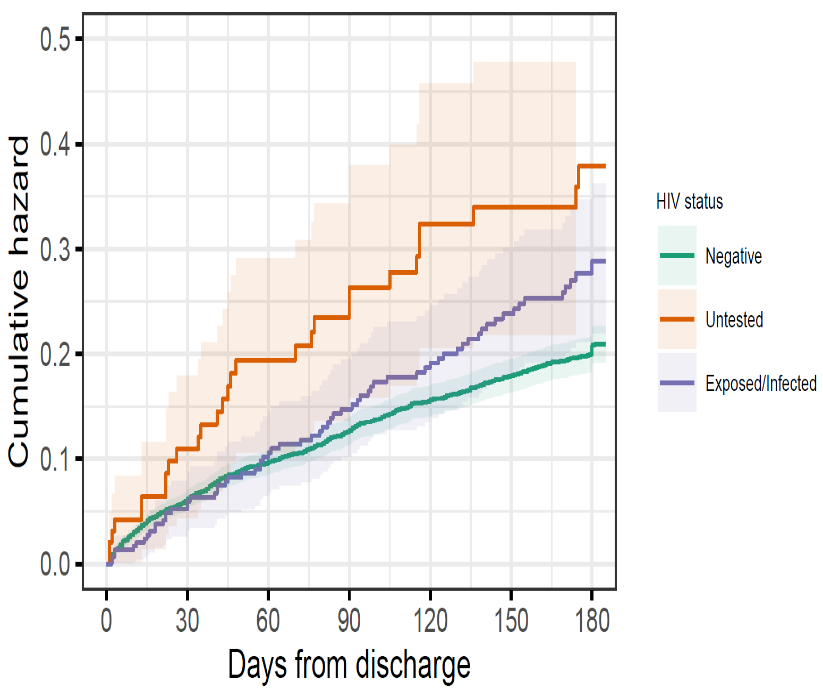 |
| C  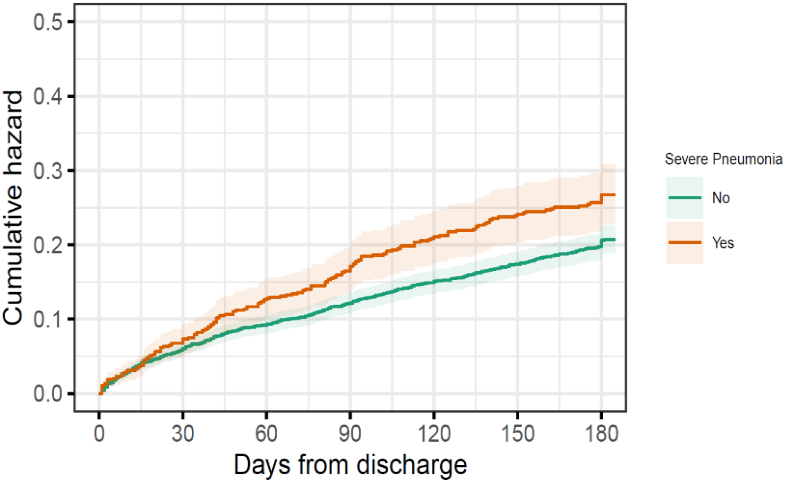 |

## Supplementary Figure 2. Cumulative hazard of readmission by admission clinical syndromes/diagnosis of: a) Chronic conditions, b) HIV, and c) Severe Pneumonia.

| 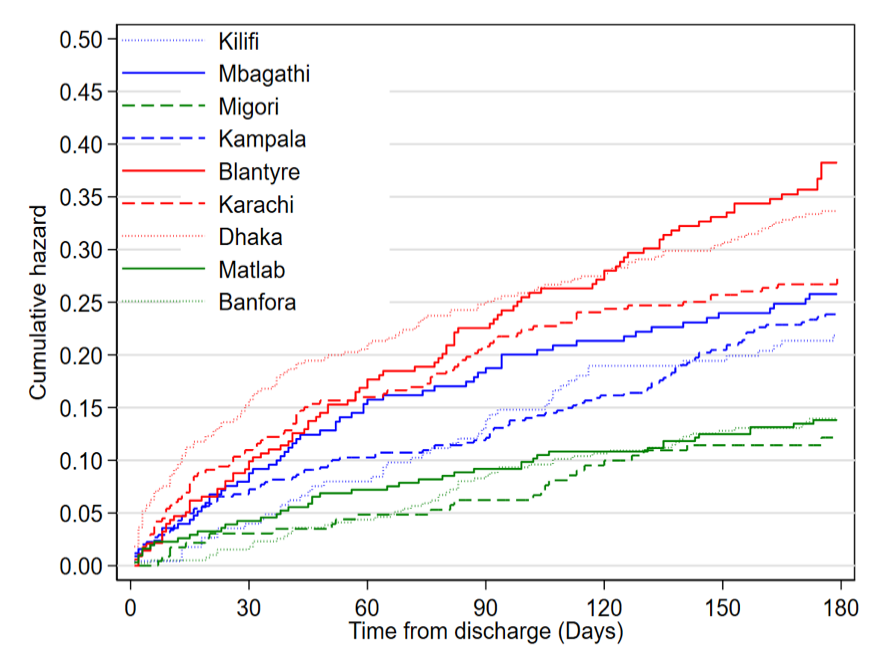 |
| --- |

## Supplementary Figure 3. Cumulative hazard of readmission by site.


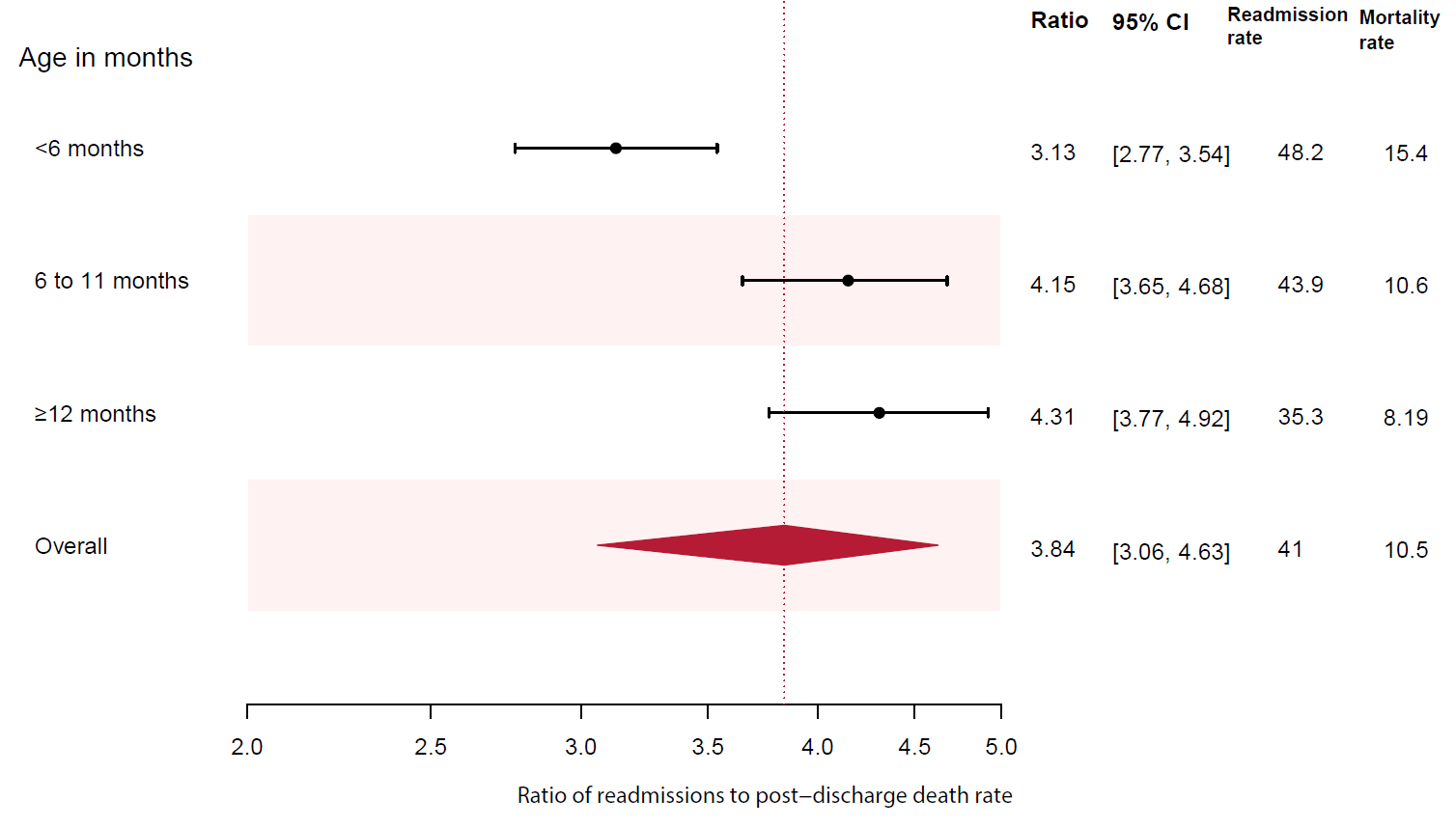
Supplementary Figure 4. Ratio of hospital readmission to post-discharge death rate by age group.

Hospital readmissions and post-discharge mortality rates are per 1000 child-years.


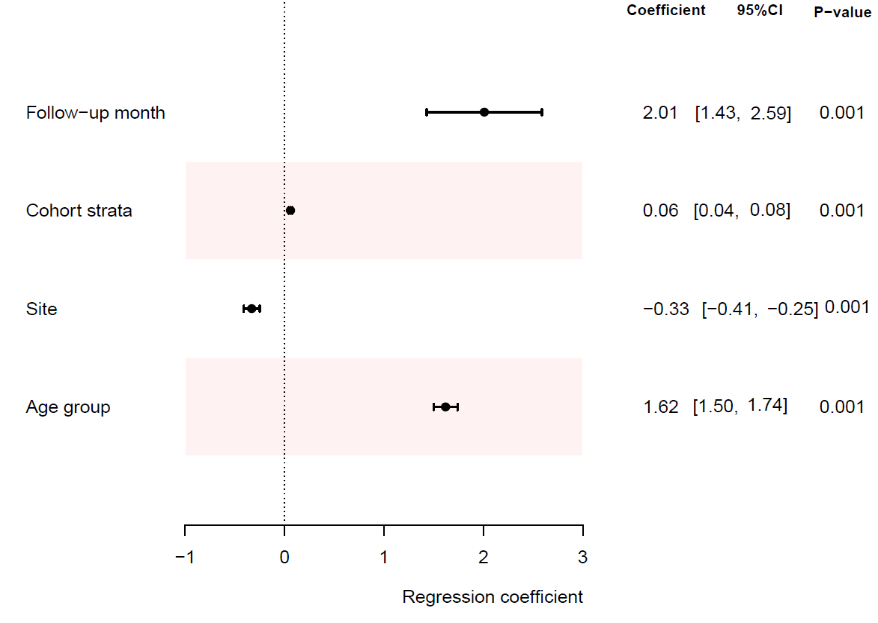


## Supplementary Figure 5. Linear regression of non-fatal hospital readmission and post-discharge death rate across selected groups.


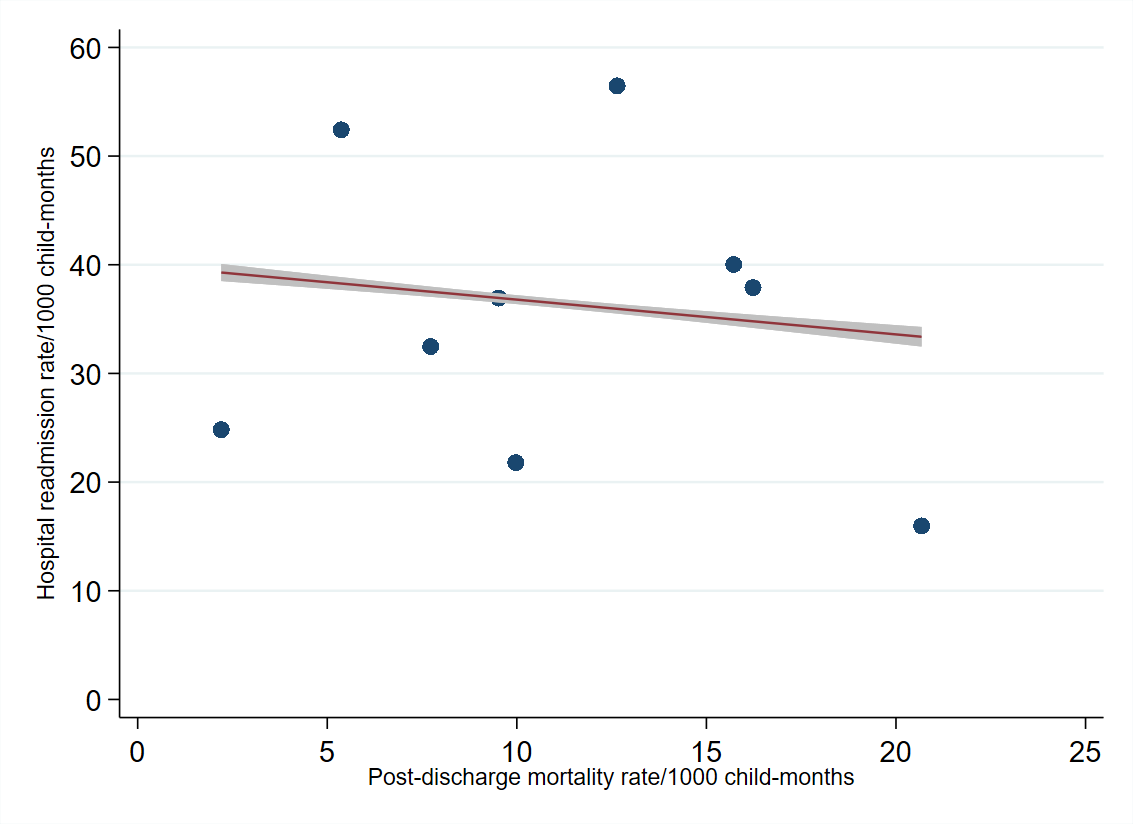


## Supplementary Figure 6. Scatter plot of hospital readmissions (fatal and non-fatal) and post-discharge mortality rates across the sites.

##
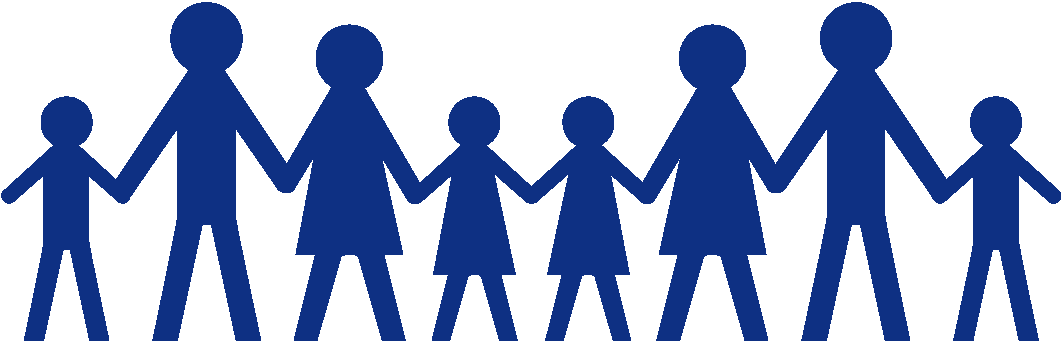


The Childhood Acute Illness & Nutrition Network

­ Hospital readmissions following acute illness among children 2-23 months old in Africa and South Asia.

The Childhood Acute Illness and Nutrition (CHAIN) Network

# **Statistical Analysis Plan**

| **Version Control** | **Update after any change** |
| --- | --- |
| **Version 1.0** | **Created by Moses Ngari 26 June 2022** |
| **Version 1.1** | **Updated by Jay & Moses 22 July 2022** |

This statistical analysis plan is prepared according to **STROBE** statement checklist for cohort studies[1].

Table of Contents

[*1.* *Background* 25](#_Toc162446886)

[*2.* *Objectives* 26](#_Toc162446887)

[*2.1.* *General objective* 26](#_Toc162446888)

[*3.* *Study Design* 26](#_Toc162446889)

[*3.1.* *Setting* 27](#_Toc162446890)

[*3.2.* *Participants* 27](#_Toc162446891)

[*Inclusion criteria* 27](#_Toc162446892)

[*Exclusion criteria* 27](#_Toc162446893)

[*Stratification* 28](#_Toc162446894)

[*Timelines* 28](#_Toc162446895)

[*3.3.* *Variables* 28](#_Toc162446896)

[*Outcomes* 28](#_Toc162446897)

[*Exposures* 28](#_Toc162446898)

[*Potential confounders and effect modifiers* 29](#_Toc162446899)

[*3.4.* *Bias* 29](#_Toc162446900)

[*3.5.* *Study Size* 30](#_Toc162446901)

[*3.6.* *Quantitative variables* 31](#_Toc162446902)

[*4.* *Statistical methods* 31](#_Toc162446903)

[*4.1.* *Participants* 31](#_Toc162446904)

[*4.2.* *Baseline characteristics* 32](#_Toc162446905)

[*4.3.* *Follow up* 32](#_Toc162446906)

[*4.4.* *Outcome data* 32](#_Toc162446907)

[*4.5.* *Main results* 32](#_Toc162446908)

[*4.6.* *Summary of analyses* 33](#_Toc162446909)

[*4.7.* *Regression models - methods* 33](#_Toc162446910)

[*Binomial regression models* 33](#_Toc162446911)

[*Survival regression models* 33](#_Toc162446912)

[*5.0* *Prespecified sub analyses* 34](#_Toc162446913)

[*References* 35](#_Toc162446914)

# Background

Although significant progress has been made in reduction of childhood mortality, in Sub-Saharan Africa and South Asia, preventable deaths remain high[2]. Most of these deaths are from infectious diseases that can be prevented by timely treatment. However, there is increasing evidence to show children are particularly vulnerable following hospital-discharge [2]. Adult patient who survive hospital admission usually have long-term sequelae, including high risk of hospital readmissions but little data is available about paediatric long-term outcomes including readmission[3-5]. There has been two previous reviews covering paediatrics hospital readmissions; a) the first review included 29 studies but only among children with asthma [6] and b) included 44 studies from developed countries only (USA, UK, Australia, Canada and Switzerland)[5].

Among children admitted to hospital in Sub-Saharan Africa and South Asia with acute illness, it is not clear how the type of illness, its severity, background comorbidities, nutritional status and treatment contribute to long-term outcomes like hospital readmission after index discharge. Following index hospital discharge, social circumstances and access to care may also be important determinants of readmission risk. In prior studies, differences in design have impaired our ability to make meaningful comparisons between sites. Understanding readmission risks after hospital discharge will help identify needs for further characterising and identify potential new interventions to deliver effective care post-discharge. Furthermore, a composite endpoint of death or readmission is commonly used in clinical trials, but we do not know whether these two outcomes have fundamentally different epidemiology, including potential biases such as access to hospital.

# Objectives

# General objective

To estimate the incidence of hospital readmission following index discharge and its associations.

**Specific objectives**

Among children aged 2 to 23 months discharged from index admission to hospital with acute illness across enrolment strata, we aim:

1. To describe incidence and case fatality of hospital readmissions.
2. To determine admission and index discharge factors associated with hospital readmission including distance to nearest hospital.
3. To compare index admission & discharge factors associated with early versus late readmissions (Early admissions are admissions ≤30 days and late are readmissions >30days following index discharge).
4. To explore differences and factors associated with hospital readmission Vs community deaths (why do some children die at home without being admitted?)
5. To compare the time patterns and risk factors for readmission with those for death (Can a composite endpoint of readmission & death vs death only be used in RCT?)

# Study Design

The CHAIN Cohort was designed as a prospective stratified cohort study. For this analysis, only children who were discharged alive will be included (N=2874). Children were on follow-up for 180 days following index hospital discharge.

# Setting

CHAIN study recruited from nine sites in low and middle-income countries (LMICs) (Bangladesh: Dhaka Hospital, Matlab Hospital, Burkina Faso: Banfora Referral Hospital, Kenya: Kilifi County Hospital; Mbagathi Sub-County Hospital, Nairobi; Migori County Hospital, Malawi: Queen Elizabeth Hospital, Blantyre, Pakistan: Civil Hospital, Karachi and Uganda: Mulago Hospital, Kampala). These sites all serve vulnerable populations and represent a range of environments, populations, access and levels of background comorbidities such as malaria and HIV.

# Participants

Eligible children were recruited at admission to hospital in three strata to ensure a spectrum of nutritional status. The comprehensive nature of data collection, sampling and follow up meant limiting the rate of enrolment to address data quality and workload. Participants were identified by choosing the first admissions from a specified day each week until the weekly quota for each stratum was met. Children were treated according to current national and international clinical guidelines.

For this analysis only a subset of children discharged alive will be included.

### Inclusion criteria

- Aged 2 to 23 months.
- Admitted to a study hospital.
- Planning to remain in the hospital catchment area for at least 6 months and willing to come for the specified follow up visits.
- Provided informed consent.
- Discharged alive.

### Exclusion criteria

- Requiring immediate resuscitation at admission defined by ongoing cardiac or pulmonary arrest or judged to be peri-arrest by the attending physician.
- Unable to tolerate oral feeds while in his/her usual state of health.
- Underlying terminal illness that in the opinion of the treating physician is likely to lead to death within 6 months (e.g., cancer, congenital heart disease).
- Diagnosed with a condition that in the opinion of the treating physician is likely to require surgery within 6 months.
- Diagnosed chromosomal abnormality (syndromically or genetically diagnosed abnormality).
- Primary reason for admission is poisoning, trauma or a surgical condition.
- Caregiver plans to move outside of the hospital catchment area within 6 months.
- Caregiver is unwilling to attend study visits.
- Previously enrolled in this study.
- Sibling currently or previously enrolled in this study.

### Stratification

Children were enrolled in three strata classified at hospitalisation using mid-upper arm circumference (MUAC):

- Severe wasting/kwashiorkor (SWK): MUAC <11.5cm (MUAC <11.0cm under 6 months old) or kwashiorkor.
- Moderate wasting (MW): MUAC 11.5 to <12.5cm at any age or MUAC 11.0 to <12.0cm under 6 months old
- Not wasted (NW): MUAC ≥12.5cm at any age or MUAC ≥12cm under 6 months old.

### Timelines

Recruitment began on 20^th^ November 2016 and ended on 31^st^ January 2019 with 180 days post index hospital discharge follow up and defaulter tracing until 31^st^ August 2019.

# Variables

### Outcomes

- Hospital readmission from date of index hospital discharge until 180 days later. Date of hospitalisation were confirmed from hospital records.
- Causes and survival of hospital readmission estimated from all available hospital records. This will be limited to readmissions in the study hospital hospitals.

### Exposures

All exposure variables to be considered were collected in a standardized Case Report Form (CRF) using study standard operation procedure (SOP)s by trained staff. The table below shows the exposure variables to be assessed in this study.

Table 1.0. **Study exposure variables.**

| **Variable** | **Type of variable** |
| --- | --- |
| Sex | Binary [male/female] |
| Age in months | Continuous [to be transformed] |
| Enrolment strata/continuous MUAC/WAZ | Ordinal [NW, MW, SW/Kwash]/continuous |
| Oedema | Binary [Yes/No] |
| Prior admission | Binary [Yes/No] |
| Length of index hospitalization | Continuous [to be transformed] |
| Discharged against medical advice | Binary [Yes/No] |
| SIRS at discharge | Binary [present/absent] |
| Chronic illness (TB, sickle cell, cerebral palsy) | Binary [present/absent] |
| HIV status | Ordinal [positive, negative, exposure, Not done] |
| Stunting at admission | Binary [present/absent] |
| Discharged on a weekend | Binary [Yes/No] |
| Common admission diagnosis (Pneumonia, diarrhoea, Malaria, Anaemia) | Binary [present/absent] |
| Small birth size | Binary [Yes/No] |
| Biological mother as primary carer | Binary [Yes/No] |
| Mother currently sick | Binary [Yes/No] |
| Caregiver education | Ordinal [None, Primary, above primary] |
| Maternal mental health score (PHQ9) | Ordinal [None, Mild/moderate, severe] |
| Caregiver working | Binary [Yes/No] |
| Assets index | Ordinal [Quintiles] |
| Household food insecurity | Ordinal [Low, medium, High] |
| Distance to study hospital and nearest hospital | Continuous [to be transformed] |
| Biochemical features at discharge (Sodium, Potassium, ALT) and haemoglobin, white blood count (WBC). | Continuous [to be transformed] |

###

### Potential confounders and effect modifiers

- Age, sex will be included as *a priori* confounders in all models, will be retained in the multivariable regression models and their effect reported.
- Sites may have unobserved/unmeasured differences in accessibility, usage, populations, treatments available, staffing, clinical syndromes profile (unmeasured confounding effects). These will be tested for and if necessary, multilevel model survival models will be constructed, accounting for non-independence of participants and effects of sites[7]. Admission hospital will be included as random intercept in the regression models.
- Since this was a stratified cohort, we will use sampling weights in all the regression models.

# Bias

Selection bias, children were purposively selected for recruitment in one of the three strata which created a non-proportional sample compared to all the admitted children. Analyses of associations with hospital readmission will be assessed by using inverse weight estimates of the probability of being selected from all admissions in the study hospitals from available site data and existing literature, and interactions tested.

Attrition and Deaths. Loss to follow up or withdrawal (LTFU) was addressed during the study by minimizing LTFU through active follow ups, phone calls and home visits for children missing their scheduled clinical visits or absconding from hospital, including after the specified end of follow up period. Bias resulting from LTFU and deaths that may be associated with strata, site or other exposures will be adjusted for in the analyses by weighting inversely to the probability of LTFU or death by site and enrolment strata giving 27 groups. Since this will be a multiple event analysis, competing risk analysis will not be feasible (deaths & LTFU can be assumed to compete with hospital readmission).

To reduce reporting bias, we collected data on hospital readmissions that occurred outside the study hospitals using hospital records brought by the caregivers during the planned visit as well as those to study hospitals. Caregivers were asked to report to the study team any time the child was re-admitted in non-study hospital.

To reduce measurement bias, we standardised clinical care across the sites, training and assessment of clinical signs and definitions. Identical anthropometry equipment was centrally purchased, scales were calibrated, and measurements were performed by two independent observers and their arithmetic mean used in analysis. Data with implausible (absolute or relative to either other cohort participants or to the same participant measured at difference time points) were referred back to sites for resolution or set to ‘missing’ where unresolvable. A summary of implausible results will be tabulated by strata, site and age.

Missing data will be handled as follows for different type of variables:

Clinical and routine laboratory test: A `missing/test not done’ category will be added such as for routine tests like infant HIV, malaria RDT or variables where pre-defined categories are useful to the analysis, such as haemoglobin. For variables used in continuous form, such as blood glucose, we may include a dummy variable for missing data. For oxygen saturation, children with unrecordable value despite efforts to measure it or were measured while in oxygen therapy will be classified as having hypoxia.

Anthropometry: For the few records missing anthropometries at discharge (weights, heights), admission values will be used where possible.

Laboratory variables: Complete blood count (haemoglobin and white blood cells) will be categorised according to WHO and paediatric sepsis guidelines[8], with a category for missing values.

# Study Size

The original CHAIN cohort study was designed to have a power of 80% to detect differences in proportion of children who would die post-discharge between non-wasted and moderately wasted children, with α=0.05 and allowing up to 10% loss to follow-up. The final version of the study protocol specified at least 2,600 children to be discharged alive and followed up post-discharge. In the actual study database, 3,101 acutely ill children were enrolled, and 2,874 children were followed post-discharge. Crude proportions of those who died post discharge were:

| Group | % died post discharge | P value for Hazards Ratio |
| --- | --- | --- |
| NW | 1.67 | Reference |
| MW | 4.09 | 0.002 |
| SW/K | 11 | <0.001 |

In this analysis, 506/2874 (18%) children had at least one hospital readmission. With 2,874 children on follow-up after index discharge, 18% risk of hospital readmission, a two-sided alpha of 0.05, the study will have at least 90% power to detect hazard ratio of ≥1.5 of hospital readmission between children with previous hospital admission compared to those not previously admitted.

# Quantitative variables

- Small birth size will be defined as either low birth weight (birth weight <2.5kg) or estimated to have been born premature (gestation age <37 weeks) since actual birthweight was unknown by parents/guardians in approximately a third of participants.
- Anthropometric z-scores will be computed using WHO 2006 references and where appropriate categorized into severe (<-3), moderate (-3 to -2) and normal (≥-2).
- Disease syndromes will be constructed following definitions used in WHO treatment guidelines.
- Length of hospital stay, age in months and other continuous variables will be accessed for normality assumption and appropriate transformation performed where necessary.
- Haemoglobin and oxygen saturation will be corrected for altitude[9]
- Systemic inflammatory response syndrome (SIRS) will be defined by two or more symptoms including hypothermia or fever, tachypnoea, tachycardia and change in white blood cells count[8].
- An asset index will be derived using principal component analysis (PCA) by including household assets ownership and housing structure variables and categorised into five quintiles[10].
- Data on caregiver’s mental health will be summarized into a total score by summing the responses of all the nine questions in the PHQ-9[11]. This will further be categorised into three groups indicating the degree of features of depression for analysis: 1) minimal (0 to 9), 2) moderate (10 to 14) and 3) severe (≥15).
- A food insecurity variable will be derived using normalized scores and categorised into three groups; low, medium and high[12].
- Appropriate diet variable will be defined as: 1) ≥4 food groups for children >8months old, 2) exclusive breast fed and one or more food groups for children 6 to 8 months old and 3) exclusive breast fed for infants <6 months old.
- GPS location and estimates made from the population density, distance to the study hospital and nearest health facility using standard methods.

# Statistical methods

# Participants

A flow chart (Figure 1) will detail the number of hospital readmissions:

- Children included in the study at index admission (N=3101).
- Children discharged alive and followed up (N=2874)
- Children readmitted to hospital
- LTFU or died after discharge from the index admission.

# Baseline characteristics

Characteristics at index hospital admission and discharge will be described using N and proportions (Table 1) stratified by hospital readmission status. For continuous variables including age and anthropometric measurements, mean (sd) and median (IQR), depending on distribution will be reported.

# Follow up

Time to hospital readmission will be calculated from date of index discharge to date of readmission, death, LTFU or 180days later. Hospital readmission rate will be calculated per 1000 child-months.

# Outcome data

The study outcome will be hospital readmission after index discharge. Since a child can have multiple hospital admissions, the outcome will be a multiple event outcome.

# Main results

- Episodes of hospital readmissions stratified by enrolment strata.
- Hospital readmission rates and rate ratios by enrolment strata adjusted for age, sex, with site as random effect, weighted for observed LTFU and deaths.
- An overall estimate for readmission for the ward population by applying the weights to simulate an unbiased population.
- Monthly hospital readmissions rates with 95% confidence intervals.
- Hospital readmission rates stratified by recruiting hospital (site).
- Table of hospital readmissions diagnosis among children admitted to study hospitals only.
- Table of reasons for hospital readmissions following condition-specific index admissions.
- Multilevel survival regression models to determine factors associated with hospital readmissions.
- Appropriate binomial regression model to examine factors associated with early versus late readmissions with site as random effect, weighted for observed LTFU and deaths.
- Appropriate binomial regression model to identify factors associated with all hospital readmissions Vs community deaths with site as random effect, weighted for observed LTFU.
- Appropriate multilevel survival regression models for i) deaths and ii) hospital readmissions and a table or forest plots ranking the hazard ratios to identify differences between hospital readmission and deaths.

# Summary of analyses

Hospital readmissions will be reported as episodes because a child can have multiple readmissions. Overall hospital readmissions incidence rate will be reported per 1000 child-months and monthly. Reasons for hospital readmissions will be reported as frequency and their respective proportions. Reasons for hospital readmissions stratified by the condition-specific at index admission will also be reported. Since data on reasons for hospital readmission in a non-study hospital are not available, reporting of reasons for readmissions will be restricted to hospital readmissions in study hospitals. Early readmissions will be defined as hospital readmissions within 30 days after index discharge while late readmissions will be readmissions after 30days following index discharge. We will explore differences and features associated with early vs late readmissions using appropriate binomial regression model with site as random effect and inversely weighted for sampling weights and LTFU/post-discharge deaths. Appropriate binomial regression model will be used to assess features association with all hospital readmissions vs community deaths. Timing of either community death or hospital readmissions will be reported as median days [interquartile range] and compared using log-rank test. A multilevel parametric survival regression model will be used to explore features associated with time to hospital readmission (s) with both site and individual child as random effect and inverse weighted for sampling and LTFU/death weights.

# Regression models - methods

### Binomial regression models

To explore features associated with early vs late hospital readmissions, a generalized linear model (GLM) with a logit link and binomial family of distribution will be used because of its robustness. The GLM will include site as a random effect and sampling inverse weights. A similar GLM model will be used to explore features associated with all hospital readmissions vs community deaths. The measure of effect from the GLM models will be regression coefficients transformed into risk ratios (95% confidence intervals). Exposures to be included in the regression models are listed on Table 1.0. The exposures were informed by a previous systematic review of factors associated with paediatric unplanned hospital readmissions[5].

### Survival regression models

A multilevel survival regression model to account for site (recruiting hospital) variation, multiple outcomes (hospital readmissions) per subject and sampling weights will be adopted. LTFU and post-discharge deaths could preclude a chance of observing hospital readmission and were thus considered to be informatively censored. A competing risk survival regression model that would have included LTFU and post-discharge deaths as competing events does not work with multiple failures per subject. Probability of LTFU and post-discharge deaths will be computed and included in the survival regression models as inverse weights to account for the possible informative censoring.

A mixed-effects parametric survival-time model will be considered with both recruiting site and subject record included as random effects and weighted for both sampling weights and inverse probability of LTFU or post-discharge deaths. Different parametric survival time distributions will be assessed for their fit to our data using predicated Cox–Snell residuals, the Akaike information criterion (AIC) and the Log likelihood test. Proportional hazard assumption will be tested using the Schoenfeld residuals method and where evidence of violation is found, accelerated failure-time models will be fixed and time ratios (TR) rather Hazard ratios (HR) reported.

Features will be selected for inclusion in the multivariable regression models using backward stepwise method where all the exposures will be put in the multivariable model and only those with P<0.1 will be retained. Any a prior confounder (age, sex) will not be subjected to feature selection criteria.

We will assess the multivariate regression models’ goodness of fit using bootstrapped area under the receiver operating curves (AUROC) with a probit model, resampled 1000 times with replacement.

# Prespecified sub analyses

1. To explore why some child die in the community without seeking health care in hospital, appropriate binomial regression models will be used to explore factors associated with all hospital readmissions Vs community death. Time to either community death or hospital readmissions will be reported as median days [interquartile range] and compared using log-rank test.
2. To test whether hospital readmission & deaths can be used as composite endpoint in RCTs Vs deaths only, multivariable regression models including same exposures will be performed and compared using a) a table ranking hazard ratios for all exposures in the two models, b) comparing the overall multivariable models performance using AUCs and c) plotting the ratio of deaths to readmissions at each month to understand the timing of deaths and readmissions.

# References

1. von Elm E, Altman DG, Egger M, Pocock SJ, Gotzsche PC, Vandenbroucke JP, Initiative S: **The Strengthening the Reporting of Observational Studies in Epidemiology (STROBE) statement: guidelines for reporting observational studies**. *J Clin Epidemiol* 2008, **61**(4):344-349.

2. Wiens MO, Kissoon N, Kabakyenga J: **Smart Hospital Discharges to Address a Neglected Epidemic in Sepsis in Low- and Middle-Income Countries**. *JAMA Pediatr* 2018, **172**(3):213-214.

3. Braet A, Weltens C, Sermeus W: **Effectiveness of discharge interventions from hospital to home on hospital readmissions: a systematic review**. *JBI Database System Rev Implement Rep* 2016, **14**(2):106-173.

4. van Walraven C, Bennett C, Jennings A, Austin PC, Forster AJ: **Proportion of hospital readmissions deemed avoidable: a systematic review**. *CMAJ* 2011, **183**(7):E391-402.

5. Zhou H, Roberts PA, Dhaliwal SS, Della PR: **Risk factors associated with paediatric unplanned hospital readmissions: a systematic review**. *BMJ Open* 2019, **9**(1):e020554.

6. Chung HS, Hathaway DK, Lew DB: **Risk factors associated with hospital readmission in pediatric asthma**. *J Pediatr Nurs* 2015, **30**(2):364-384.

7. Martins A, Aerts M, Hens N, Wienke A, Abrams S: **Correlated gamma frailty models for bivariate survival time data**. *Stat Methods Med Res* 2019, **28**(10-11):3437-3450.

8. Comstedt P, Storgaard M, Lassen AT: **The Systemic Inflammatory Response Syndrome (SIRS) in acutely hospitalised medical patients: a cohort study**. *Scand J Trauma Resusc Emerg Med* 2009, **17**:67.

9. Sullivan KM, Mei Z, Grummer-Strawn L, Parvanta I: **Haemoglobin adjustments to define anaemia**. *Trop Med Int Health* 2008, **13**(10):1267-1271.

10. Vyas S, Kumaranayake L: **Constructing socio-economic status indices: how to use principal components analysis**. *Health Policy Plan* 2006, **21**(6):459-468.

11. Kroenke K, Spitzer RL, Williams JB: **The PHQ-15: validity of a new measure for evaluating the severity of somatic symptoms**. *Psychosom Med* 2002, **64**(2):258-266.

12. Cafiero C, Melgar-Quinonez HR, Ballard TJ, Kepple AW: **Validity and reliability of food security measures**. *Ann N Y Acad Sci* 2014, **1331**:230-248.
